# Diversity and Expression of Bacterial Metacaspases in an Aquatic Ecosystem

**DOI:** 10.3389/fmicb.2016.01043

**Published:** 2016-07-06

**Authors:** Johannes Asplund-Samuelsson, John Sundh, Chris L. Dupont, Andrew E. Allen, John P. McCrow, Narin A. Celepli, Birgitta Bergman, Karolina Ininbergs, Martin Ekman

**Affiliations:** ^1^Science for Life Laboratory, Department of Ecology, Environment and Plant Sciences, Stockholm UniversitySolna, Sweden; ^2^Science for Life Laboratory, Department of Biology and Environmental Science, Linnaeus UniversitySolna, Sweden; ^3^Microbial and Environmental Genomics, J. Craig Venter InstituteSan Diego, CA, USA

**Keywords:** metacaspases, caspases, bacterial communities, metagenomics, metatranscriptomics, Baltic Sea, Cyanobacteria, *Nodularia spumigena*

## Abstract

Metacaspases are distant homologs of metazoan caspase proteases, implicated in stress response, and programmed cell death (PCD) in bacteria and phytoplankton. While the few previous studies on metacaspases have relied on cultured organisms and sequenced genomes, no studies have focused on metacaspases in a natural setting. We here present data from the first microbial community-wide metacaspase survey; performed by querying metagenomic and metatranscriptomic datasets from the brackish Baltic Sea, a water body characterized by pronounced environmental gradients and periods of massive cyanobacterial blooms. Metacaspase genes were restricted to ~4% of the bacteria, taxonomically affiliated mainly to Bacteroidetes, Alpha- and Betaproteobacteria and Cyanobacteria. The gene abundance was significantly higher in larger or particle-associated bacteria (>0.8 μm), and filamentous Cyanobacteria dominated metacaspase gene expression throughout the bloom season. Distinct seasonal expression patterns were detected for the three metacaspase genes in *Nodularia spumigena*, one of the main bloom-formers. Clustering of normalized gene expression in combination with analyses of genomic and assembly data suggest functional diversification of these genes, and possible roles of the metacaspase genes related to stress responses, i.e., sulfur metabolism in connection to oxidative stress, and nutrient stress induced cellular differentiation. Co-expression of genes encoding metacaspases and nodularin toxin synthesis enzymes was also observed in *Nodularia spumigena*. The study shows that metacaspases represent an adaptation of potentially high importance for several key organisms in the Baltic Sea, most prominently Cyanobacteria, and open up for further exploration of their physiological roles in microbes and assessment of their ecological impact in aquatic habitats.

## Introduction

Caspases are proteases that initiate targeted protein degradation and the execution of apoptotic programmed cell death (PCD) pathways in metazoan tissues (Elmore, 2007; McIlwain et al., 2013). Caspases may also take part in non-PCD activities, such as cell differentiation, cell proliferation, and the immune response, hence playing a fundamental role in life and death of animal and human cells (Kuranaga and Miura, 2007). This diversity of physiological functionality is reflected in the numerous types of caspases recognized, all characterized by their highly conserved catalytic cysteine-histidine dyad at the active site (McLuskey and Mottram, 2015).

At the turn of the century, proteins displaying sequence homology to caspases were identified in non-metazoan eukaryotes such as protists, plants, and fungi, but also in bacteria (Aravind et al., 1999; Uren et al., 2000). Such caspase homologs are classified and named according to their sub-domain organization, activation mechanism, and proteolytic target specificity (Asplund-Samuelsson, 2015). Here, we will refer to these putative proteases as “metacaspases.” Recently, computational analysis of fully sequenced prokaryotic genomes showed that ~19% of sequenced bacteria carry metacaspase genes, in particular Alphaproteobacteria, Deltaproteobacteria, and Cyanobacteria (Asplund-Samuelsson et al., 2012). The highest metacaspase frequency (up to 12 metacaspase genes per genome) was found in filamentous multicellular Cyanobacteria capable of cell differentiation, phylogenetically belonging to Clade 1 Cyanobacteria (Larsson et al., 2011), such as the genera *Trichodesmium, Nostoc*, and *Anabaena*. This is in contrast to small, marine, and globally wide-spread unicellular cyanobacteria of the genera *Synechococcus* and *Prochlorococcus*, which largely lack these genes (Bidle and Falkowski, 2004; Jiang et al., 2010; Asplund-Samuelsson et al., 2012). Likewise, other bacteria commonly carry only one or two metacaspase genes, if any.

Today, studies provide support for a role of metacaspases related to cell death, e.g., in yeast (Madeo et al., 2002) and *Arabidopsis* (Coll et al., 2010), as well as non-PCD activities, such as stress response (Richie et al., 2007) and virulence mediation (Proto et al., 2011). Based on their protein domain architectures, bacterial metacaspases have also been suggested to take part in basic cellular processes, including protein modification, PCD, signaling, and a range of enzymatic activities (Asplund-Samuelsson et al., 2012). Nevertheless, the presence, identity, and physiological role of metacaspases in the prokaryotic realm remains largely unknown.

Bacterial PCD is known from representatives across several phyla (Engelberg-Kulka et al., 2006; Tanouchi et al., 2013; Bayles, 2014). In Cyanobacteria, processes resembling PCD have been observed in *Anabaena* sp. PCC 7120 during salt stress (Ning et al., 2002), in the unicellular bloom-former *Microcystis* (Ross et al., 2006; Sigee et al., 2007; Ding et al., 2012), and in marine *Trichodesmium* (Berman-Frank et al., 2004; Bidle, 2015), where PCD is linked to bloom dynamics. Various modes of PCD have also been morphologically observed in the *Nostoc*-type species living in symbiosis with the waterfern *Azolla* (Zheng et al., 2013). Further, stress-related (e.g., nutrient and oxidative stress) PCD events were found to correlate with metacaspase gene expression in the marine cyanobacterium *Trichodesmium* (Bar-Zeev et al., 2013) and caspase-like protein activity in *Microcystis* (Ross et al., 2006; Ding et al., 2012). A similar connection was observed between stress, PCD and expression of metacaspase-like proteins in the plant pathogen *Xanthomonas* (Wadhawan et al., 2010) and in *Echerichia coli* K-12 substrain MG1655 (Wadhawan et al., 2013). Environmental stresses such as iron starvation and osmotic or heat shock may also cause the unicellular eukaryotic phytoplankton *Thalassiosira* and *Dunaliella* to undergo PCD in conjunction with activation of metacaspases (Bidle and Bender, 2008; Jiménez et al., 2009).

Like caspases, metacaspases show proteolytic capability (Vercammen et al., 2007; Tsiatsiani et al., 2011, 2013; Klemenčič et al., 2015) and carry the highly conserved catalytic cysteine-histidine dyad motif (McLuskey and Mottram, 2015), but available data indicate that their cleavage specificity (arginine and/or lysine) differs from that of caspases (aspartate; Tsiatsiani et al., 2011; Klemenčič et al., 2015; McLuskey and Mottram, 2015). Previous studies investigating bacterial PCD have however often utilized cleavage of aspartate-containing substrates, e.g., Ac-DEVD-AMC (Gautam and Sharma, 2002; Wadhawan et al., 2010), Z-DEVD-AFC (Berman-Frank et al., 2004, 2007), Z-DEVD-R110 (Ross et al., 2006), FITC-DEVD-FMK (Ding et al., 2012), or IETD-AFC (Bar-Zeev et al., 2013), as indicators. The proteolytic activity reported in these studies thus suggests either a greater range of substrate specificity than currently recognized in metacaspases, or that the assays detect proteolytic processing by other enzymes (Asplund-Samuelsson, 2015). Despite the multitude of bacterial metacaspases now recognized, only one has been experimentally characterized (Klemenčič et al., 2015), highlighting the need to further investigate the functional distinction of metacaspases and their substrates relative to caspases. The correct classification of metacaspases remains a topic for debate (Enoksson and Salvesen, 2010; McLuskey and Mottram, 2015).

The life and death of phytoplankton profoundly affects nutrient cycling in aquatic environments through the release of organic molecules of photosynthetic origin (Bidle and Falkowski, 2004; Bidle, 2015). Whereas grazing by zooplankton entails transfer to higher trophic levels, viral lysis results in release of nutrients and particulate matter to surrounding waters (Tijdens et al., 2008). PCD is also regarded a major contributor to phytoplankton lysis, and metacaspase-based PCD may therefore be a factor in the cycling of phytoplankton-derived nutrients in aquatic ecosystems (Berman-Frank et al., 2004, 2007), a topic reviewed in Bidle (2015). PCD-based *Trichodesmium* bloom collapse was for example suggested to influence the export of carbon and nitrogen to the deep ocean (Bar-Zeev et al., 2013). The temperate semi-enclosed brackish Baltic Sea (northern Europe) suffers from severe anthropogenically induced eutrophication and anoxic bottom zones, and is characterized by yearly massive cyanobacterial blooms consisting primarily of the filamentous nitrogen-fixing strains *Aphanizomenon* sp., *Nodularia spumigena*, and *Dolichospermum* spp. (formerly known as *Anabaena*; Wacklin et al., 2009). Apart from representing an important group of primary producers, these nitrogen-fixing cyanobacteria may constitute the third largest source of new nitrogen to the Baltic after riverine load and atmospheric deposition (Degerholm et al., 2008). The blooms are thus not only an essential part of the Baltic Sea natural ecosystem but also a factor that needs to be considered in any effort to manage the eutrophication of this water body. However, many aspects of the blooms remain unknown, including the role of PCD and metacaspases in bloom dynamics and demise. Analogous to *Trichodesmium* blooms in tropical oceans, such processes may influence the export of organic C and N to deeper Baltic Sea waters and thus the extent of which the blooms contribute to the spreading of bottom anoxia.

With few exceptions, data available on the presence and role of metacaspases in bacteria are derived from analyses of cultured isolates and microbial genomes. However, such selected organisms may not adequately represent natural environments, neither in terms of taxonomic composition, nor in their response to environmental stimuli. To considerably widen our understanding of the distribution and role of metacaspases in bacterial communities, we analyzed microbial metagenomes and metatranscriptomes retrieved from a natural aquatic setting (the Baltic Sea), sampled both along a geographical north-south transect (Dupont et al., 2014) and seasonally at a coastal station (Baltic Proper). This represents the first community-wide survey of microbial metacaspases and their expression under natural conditions.

## Materials and methods

### Sampling of baltic sea microbial communities

**Figure 1:**
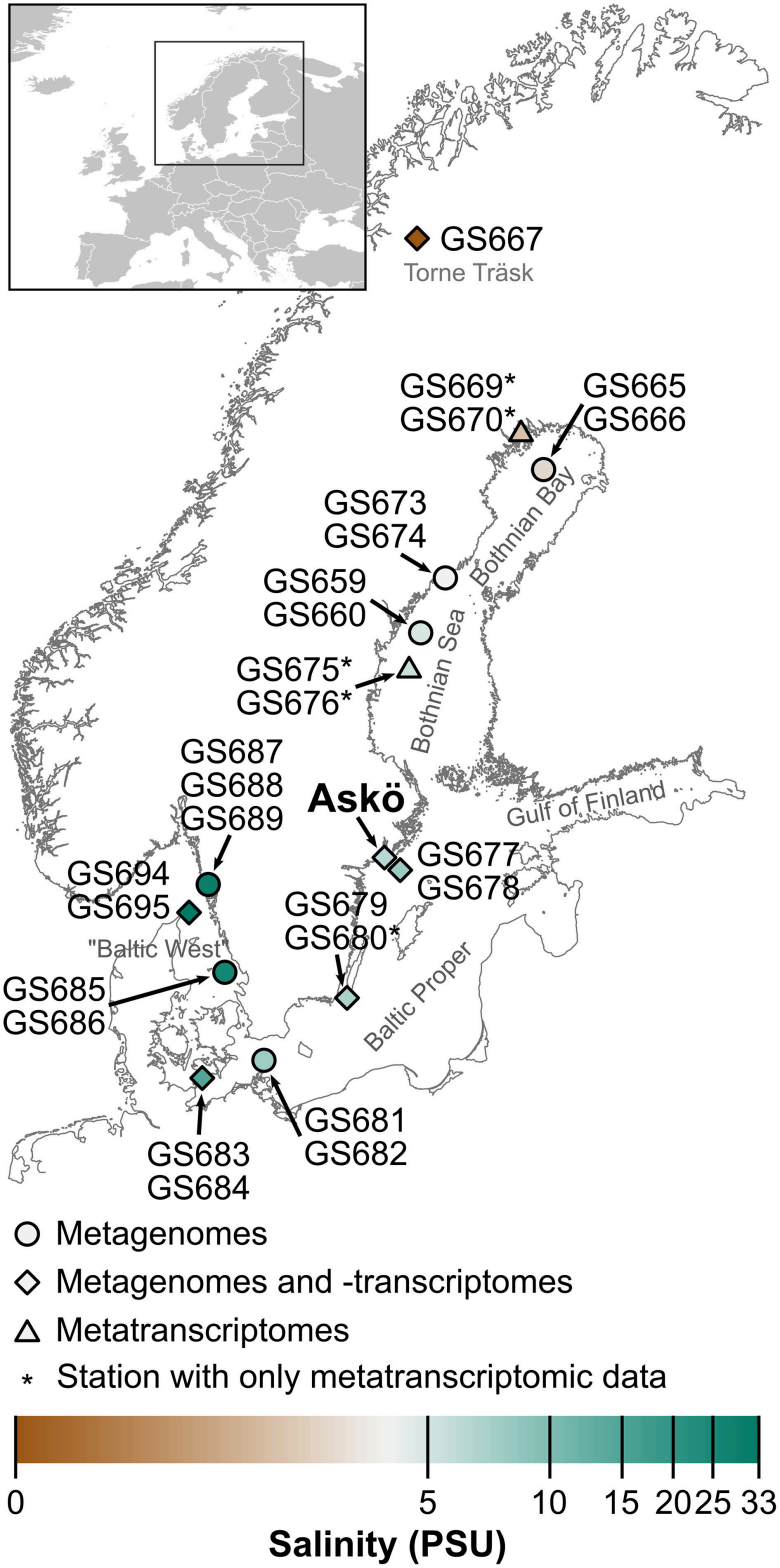
**Map of the Baltic Sea area specifying the locations of the sampling stations (GS) in the 2009 Global Ocean Sampling Baltic Sea transect (Phase I), and the time series of 2011 (Phase II) and 2012 (Phase III) off the coastal station Askö (south of Stockholm)**. The samples taken at the different stations were used for generating metagenomes, metatranscriptomes, or both, as indicated by the shape of the map marker symbols. The color of the markers show the salinity gradient inherent to this system.

Sampling was carried out in three phases. In Phase I (July 2009) microbial samples for metagenomic and metatranscriptomic (for a subset of samples) analyses were collected along a 1800 km Baltic Sea transect (Figure 1; Dupont et al., 2014). In Phases II (June–August 2011) and III (May–September 2012) seasonal samples were collected from surface waters at a coastal station off the Stockholm archipelago island Askö (Baltic Proper, 58.82°N, 17.62°E; Figure 1) for metagenomics (2011) and metatranscriptomics (2012). When sampling for metagenomics (Phases I and II) and metatranscriptomics (Phase I) water was filtered serially into four size fractions (3.0–200, 0.8–3.0, 0.1–0.8 μm, and a viral fraction 50 kDa—0.1 μm) as described in Zeigler-Allen et al. (2012) and Dupont et al. (2014). The amount of water passed through the filters was 100–200 L for Phase I and 50–150 L for Phase II. When sampling specifically for metatranscriptomics (Phase III at Askö) two liters of pre-filtered (200 μm) sea water was passed through 0.22 μm Sterivex filters. The time from water sampling to freezing sterivex filters on dry ice was ~8 min. Metatranscriptomics sampling at Askö was performed between 9 and 11 A.M.

### Sequencing, assembly, and annotation

Metagenomes were acquired from transect samples (Phase I) through 454 pyrosequencing and identification of open reading frames (ORFs) on the reads followed by APIS taxonomic classification (Dupont et al., 2014). DNA retrieved from samples from 2011 (Phase II) was sequenced with Illumina technology, yielding a total of 24 annotated metagenomic assemblies (six time points and four size fractions; Larsson et al., 2014). The reads in each sample were mapped to contigs from all assemblies using bowtie2 (v. 2.2.3). Coverage information was then calculated for each contig using bedtools (v. 2.17.0) histograms.

The Phase I and II metagenomic samples constitute previously partly analyzed datasets (Dupont et al., 2014; Larsson et al., 2014), while this study introduces the Phase I and III metatranscriptomes. For the latter, filters were frozen and kept on dry ice for shipping and stored in the laboratory at −80°C. RNA was purified from filters using the Trizol reagent (Life Technologies; Carlsbad, CA) and treated with DNase (Qiagen, Valencia, CA, USA) and cleaned with the RNeasy MinElute Kit (Qiagen, Valencia, CA, USA). RNA quality was analyzed with on a 2100 Bioanalyzer with Agilent RNA 6000 Nano Kits (Agilent Technologies, Santa Clara, CA, USA) and quantified using Qubit Fluorometric Quantification system (ThermoFisher, Waltham, MA, USA).

Total mRNA transcriptome libraries were made with ScriptSeq v2 RNA-Seq kit (Illumina Inc., San Diego, CA, USA) using subtractive hybridization of rRNAs with either antisense rRNA probes (Stewart et al., 2010), recovered via PCR from a mixture DNA obtained from 0.1, 0.8, and 3.0 μm filters from the 13 samples from the Baltic transect (Phase I, 2009), or using Ribo-Zero bacterial and plant rRNA removal kits (Illumina Inc., San Diego, CA, USA) on the 11 samples collected at Askö (Phase III, 2012) comprising this study.

Two-hundred and fifty nanogram of total community RNA was used for subtractive hybridization. Multiple rounds of subtractive hybridization of rRNAs were used to obtain at least 30 ng of rRNA deplete total RNA. rRNA subtracted total RNA quality was analyzed on a 2100 Bioanalyzer with Agilent RNA 6000 Pico Kits (Agilent Technologies, Santa Clara, CA, USA). Five nanogram of rRNA deplete total RNA was used for input into the ScriptSeq v2 RNA-Seq kit (Illumina Inc., San Diego, CA, USA) following the manufactures protocol. One-hundred and seven copies of ArrayControl RNA Spikes (Life Technologies, Carlsbad, CA, USA) were added to each sample prior to ScriptSeq amplification. Ampure XP beads (Beckman Coulter) were used for cDNA and final library purification.

Library quality was analyzed on a 2100 Bioanalyzer with Agilent High Sensitivity DNA Kits (Agilent Technologies, Santa Clara, CA, USA). Libraries were quantified using a standard Illumina Library quantification kit (KAPA Biosystems) and pooled in an equimolar fashion in three pools, which were run in three lanes of paired-end sequencing via Illumina HiSeq 2000.

Metatranscriptomic sequences were quality trimmed and filtered to remove Illumina primers and rRNA using Ribopicker (Schmieder et al., 2012) prior to assembly and read mapping. Reads were assembled into transcript contigs using CLC Assembly Cell (CLC bio). ORFs were called using FragGeneScan (Rho et al., 2010), and reads were then mapped back to the assembled transcript ORFs using CLC. In total, the dataset consists of 432,494 ORFs and 321,765,424 mapped reads. ORFs were annotated by blastp vs. PhyloDB (Dupont et al., 2015) and HMMER v. 3.1b2 hmmscan (http://hmmer.org) with the Pfam protein families database (Finn et al., 2014) and TIGRFAMs (Selengut et al., 2007).

### Identification and characterization of metacaspase and *recA* sequences

Metacaspases were identified using a bacterial metacaspase-specific Hidden Markov Model (HMM) (Asplund-Samuelsson et al., 2012) with hmmsearch (best 1 domain *E* ≤ 0.01 and score/bias ≥ 10) on translated ORF sequences in the metagenomes (HMMER v. 3.0) and metatranscriptomes (HMMER v. 3.1b1). Specificity was ensured by annotating the identified sequences with hmmscan vs. Pfam 27.0 as previously described (Asplund-Samuelsson et al., 2012), accepting both caspase and bacterial metacaspase hits. Pfam domain architectures were obtained concurrently, which also incorporate transmembrane helices identified by TMHMM (v. 2.0c; Krogh et al., 2001). Identical metacaspase transcript ORFs were detected with cdhit-est (v. 4.6).

Normalization for varying sequencing depth and genome sizes in different metagenomic samples is possible by dividing the abundance of the gene of interest with that of a single-copy gene such as *recA*. The *recA* gene may also be used for taxonomic composition analysis (Liu et al., 2012). *recA* sequences in the Phase I and Phase II metagenomes were identified using the same approach as the one used for metacaspases, but with the Pfam *recA* HMM (PF00154) in the initial hmmsearch.

### Genes on metagenomic contigs from 2011

Metacaspase sequences identified in the metatranscriptomes (Phases I and III) were matched to metagenome contigs (Phase II, 2011) via blastn, followed by CLUSTALW (v. 2.1) pairwise alignment and visual inspection of the alignments. These and other metagenome contigs containing metacaspase genes were clustered at 99% identity with cdhit-est (v. 4.6), leaving a set of contigs representing the clusters. ORFs on the contigs were subjected to the Pfam 27.0 hmmscan (HMMER v. 3.1b1) annotation procedure described above. Furthermore, the sequences were submitted to the KEGG Automated Annotation Server for KEGG KO ID assignment, using the standard prokaryotic representative gene set and the single-directional best hit method.

### Taxonomic classification of metacaspase and *recA* sequences

UniProt proteomes (http://www.uniprot.org/help/proteome) were subjected to metacaspase and *recA* queries using the method described above. The identified caspase/metacaspase or *recA* sequences were aligned to the corresponding HMM using hmmalign with the trim option. Sequences identical after alignment were reduced to one representative, which was assigned taxonomic information corresponding to the lowest common ancestor (based on the NCBI taxonomic hierarchy) of the identical sequences. The alignments were used to construct phylogenetic reference trees via the approximately maximum-likelihood method of FastTreeMP (v. 2.1.7 SSE3) at default settings (Price et al., 2010). The alignment, phylogenetic tree and data from the NCBI Taxonomy database (downloaded on September 9th 2014) were combined to create a pplacer (v. 1.1.alpha16) reference package. Baltic Sea metacaspase or *recA* sequences were then aligned to the corresponding HMM using hmmalign with the trim option and placed on the reference trees using pplacer followed by taxonomic classification with the pplacer guppy tool. The level of assignment, here selected at likelihood 0.95, ranges from strain at best up to the root of the NCBI taxonomic hierarchy at worst. The pplacer reference packages are provided as Data Sheets S1, S2.

### Estimating the average frequency of bacteria with metacaspase genes

The expected number of metacaspase genes per genome, in organisms that possess metacaspases, is available for different taxonomic groups (Asplund-Samuelsson et al., 2012). Metacaspase sequence counts were aggregated per taxonomic group (i.e., class for Proteobacteria and phylum for other bacteria) in the Phase I transect metagenomes (2009) and divided by the corresponding expected number of metacaspases per genome. The sum of these values was then divided by the total number of *recA* genes in the transect metagenomes, thereby estimating the percentage of sampled Baltic Sea bacteria that carried metacaspase genes. Proteobacteria and bacteria of unknown origin were excluded, as the expected number of metacaspases per genome is not available for these unknown groups.

### Bacterial metacaspase prevalence in different size fractions and groups

Metacaspase gene abundance (the ratio of metacaspase to *recA*, when non-zero, or to ORFs for the group) and expression level (metacaspase transcript ORF read counts divided by the total read count of all transcript ORFs in the group) was calculated for bacteria, non-cyanobacterial bacteria, and Cyanobacteria in each non-viral sample along the transect (Phase I). This facilitated comparisons between the size fractions.

Metacaspase gene abundance was also calculated for individual bacterial groups along the transect (Phase I metagenomes). The procedure entailed division of the total number of metacaspase sequences by the total *recA* or ORF count per group in metagenomes from all non-viral size fractions at each sampling station (pooling of the size fractions limited the incidence of zero *recA* counts, which cannot be used for normalization). The values obtained allowed comparison of abundances between groups as well as comparison of abundances within groups on a per genome (normalization vs. *recA*) or per gene (normalization vs. ORF counts) basis.

Metacaspase gene expression level was also calculated for each bacterial group in every metatranscriptomic sample (Phases I and III).

Gene abundances or expression levels were compared between size fractions or between bacterial groups using Kruskal-Wallis tests followed by *post-hoc* pairwise Mann-Whitney tests (R functions “kruskal.test” and “pairwise.wilcox.test”). Multiple testing correction was applied through the Bonferroni method, i.e., the significance level (*p* < 0.05) was adjusted through division by the number of tests in each set of pairwise comparisons. Only finite values were included in this analysis.

### Gene Co-expression analysis in *Nodularia spumigena*

Metatranscriptome ORFs were clustered at 100% sequence identity with cdhit-est (v. 4.6) and a representative for each cluster was chosen arbitrarily. Read counts for identical sequences were summed and granted to the representative. Cluster representative ORFs annotated as *Nodularia spumigena* CCY9414 (using blastp vs. PhyloDB; see Section Sequencing, Assembly, and Annotation) were considered the *in situ* transcriptome of this organism. The first sample of the Phase III summer season, 23 May 2012, was discarded because of the low absolute read count numbers for *N. spumigena* and the resulting loss of resolution (Figure S1). The ensuing analysis focused on the remaining 10 samples in the Phase III time series. The total number of mapped reads for *N. spumigena* transcripts in each sample was noted, followed by removal of transcripts with a total count below 200 or more than three zero counts throughout the season. Reads per kilobase and million mapped reads (RPKM) normalization was then applied. The RPKM values were inverse hyperbolic sine transformed (Burbidge et al., 1988) using the R function “asinh” at default settings to prevent skewing by highly expressed genes.

Expression pattern clusters were identified through hierarchical clustering based on Pearson correlation distance (R functions “hclust” and “corDist”). The resulting tree was cut into the number of clusters that yielded the highest average silhouette width (R function “silhouette”).

*N. spumigena* ORFs were assigned KEGG KO IDs using the KEGG Automated Annotation Server, a set of 40 cyanobacterial reference genomes and the single-directional best hit method. The functional composition, in terms of KEGG orthology category counts, of each cluster containing metacaspase genes was compared to the union of all clusters, i.e., the average functional composition, through a chi-square test with Monte Carlo simulated *p*-values (100,000 iterations; R function “chisq.test”). Note that not all KEGG KO IDs are associated with an orthology category, that not all genes have a KEGG KO annotation and that one KEGG KO can be associated with multiple orthology categories. The following KEGG orthology categories were not included in the analysis as they lack relevance to cyanobacterial physiology: “Cancers,” “Digestive system,” “Endocrine and metabolic diseases,” “Endocrine system,” “Immune diseases,” “Immune system,” “Infectious diseases,” “Nervous system,” and “Neurodegenerative diseases.” Categories not found in any cluster with metacaspase genes were combined into the “Other” category consisting of “Cell motility,” “Drug resistance,” and “Transport and catabolism.”

Transcript ORFs annotated as microcystin genes *mcyD, mcyE*, and *mcyJ* by KEGG (K16128, K16129, and K16134, respectively) present in *N. spumigena* expression pattern clusters also including metacaspase transcripts were compared to the nodularin synthesis gene cluster of *N. spumigena* NSOR10 (GenBank accession AY210783.2; Moffitt and Neilan, 2004) by performing a blastn v. 2.2.28+ search.

### Correlation of *Nodularia spumigena* metacaspase expression to environmental variables and a proxy for population size

Environmental data samples (urea, DOC, DOP, DPA, N/P ratio, PO${}_{4}^{3-}$, silica, TDN, TP, NH${}_{4}^{+}$, NO${}_{2}^{-}$, and NO${}_{3}^{-}$) were collected concurrently with the Phase III sampling and later analyzed at Virginia Institute of Marine Science (http://web.vims.edu/admin/asc/). Temperature, salinity, and oxygen were measured at the sampling site using a combined hand-held probe, while pH was measured with a pH electrode in the lab within 1 h of sampling. The data were supplemented with STRÅNG irradiation data (http://strang.smhi.se/) as means of the 3 days leading up to and including the sampling date (UV, global and direct irradiation, sunshine duration, and photosynthetic photon density). The environmental data were also supplemented with the total expression of *N. spumigena* compared to all mRNA in each sample, based on read counts, corresponding to relative population size.

The *N. spumigena* metacaspase expression patterns were subjected to correlation testing, linear modeling, redundancy analysis, and partial least squares regression vs. the environmental parameters. For visualization, a subset of the environmental data was scaled (R function “scale”) and reduced to its two principal components (R function “prcomp”). The metacaspase expression patterns were then correlated to this reduced representation of the environment.

## Results

The present study on metacaspases in the Baltic Sea is based on three sets of samples. The first set (Phase I) consists of metagenomes and metatranscriptomes collected in 2009 along a transect through the Baltic Sea, the second (Phase II) of metagenomes collected weekly during the summer season of 2011 at a single sampling site (Askö) in the central Baltic Sea, and the third (Phase III) consists of metatranscriptomes collected seasonally at the Askö sampling site in 2012. Below the results from analyzing these samples are structured as follows; (1) Phase I metagenomes, (2) Phase II metagenomes, and (3) Phase I and III metatranscriptomes.

### Analysis of spatial genetic data (phase I metagenomes)

The first stage of this survey of metacaspase genes in the Baltic Sea investigates patterns discernible in microbial metagenomes acquired along a north-south transect of the Baltic Sea (Phase I; Figure 1). The dataset comprises 21 stations with size fractionated samples: 3.0–200, 0.8–3.0, 0.1–0.8 μm, and a viral fraction. Results (gene hits) acquired from the viral metagenomes are only included in Table S1. Sequencing data is available via iMicrobe project CAM_P_0001109.

#### The baltic sea bacterial communities

**Figure 2:**
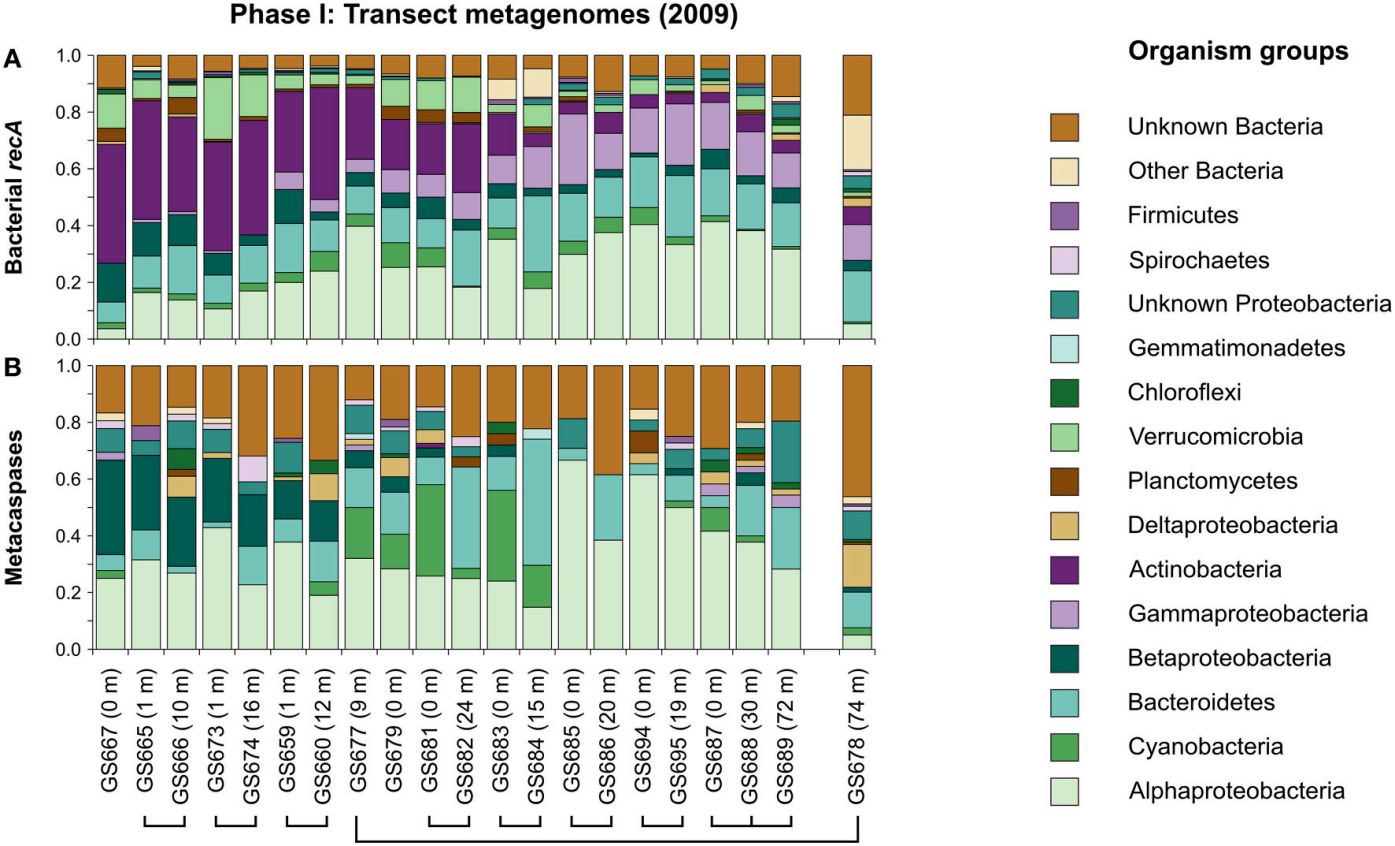
**Taxonomic distribution of bacterial ***recA*** (A) and metacaspase genes (B) in metagenomes derived along the 2009 Baltic Sea transect (Phase I; 21 stations; Dupont et al., 2014)**. The relative proportion of sequences from each sampling station (GS number) is displayed (the three non-viral size fractions combined). The taxonomic distribution at Landsort Deep (GS678) is displayed separately as this station represents an outlier (e.g., due to a low-oxygen environment). Sampling stations at the same geographical location (see Figure 1), but at different depths (shown within parentheses), are connected with lines.

In order to provide background information for the taxonomic affiliation of the metacaspases, the taxonomic composition of the Baltic Sea bacteria was first assessed throughout the whole sampling transect (Phase I; Figure 1). The strong salinity gradient (0–35 PSU) has a profound impact on the taxonomic distribution of microbes (Figure 2A), as previously shown both by shotgun metagenomics (Dupont et al., 2014) and 16S rRNA gene sequencing (Herlemann et al., 2011) of the Baltic bacterial communities. As seen in Figure 2A, using *recA* as the marker gene, Actinobacteria dominated limnic (Lake Torne Träsk) and low salinity parts of the transect in the north (Bothnian Bay), giving way for Alpha- and Gammaproteobacteria as salinity increased toward the southern “Baltic West” marine waters. Water depth did not influence the community composition patterns markedly, except for in the significantly different Landsort Deep offering hypoxic conditions (GS678 in Figures 1, 2).

The *recA* sequences identified in the Phase I metagenomes are presented in Table S1 and Data Sheet S3.

#### Geographical and taxonomic distribution of metacaspase genes

Bacterial metacaspase genes were detected at all 21 sampled locations, defined by geographical position and depth, throughout the Phase I transect (Figure 2B). The total of 901 identified genes (Table 1) corresponds to 0.16%0 of all 5,523,683 bacterial ORFs. The relative abundance of metacaspase genes along the transect showed no apparent geographical trends (Figure S3) that could be related to gradients within the system, for example the salinity gradient (Figure 1). However, as seen in Figure S3, peaks in metacaspase gene abundance were evident in microbes inhabiting the Baltic Proper (stations GS677, GS679, GS681) and the southern Danish straits (stations GS683 and GS684). These peaks coincided with an elevated proportion of cyanobacterial metacaspase genes (Figure 2B).

**Table 1:** **Number of bacterial metacaspases identified in the present study and their Pfam domain architectures**.

**Domain architecture**	**Metagenomes**		**Metatranscriptomes**	**Total**
	**Transect (Phase I)**	**Seasonal (Phase II)**	**Phases I and III**	
Metacaspase	849 (57)	334 (74)	42 (12)	1225 (143)
Peptidase_C14	28 (3)	24 (5)		52 (8)
Metacaspase|oTMHi	3	2 (2)	3 (3)	8 (5)
Metacaspase|iTMHo	2	1 (1)	1 (1)	4 (2)
Peptidase_C14|oTMHi	1			1
oTMHi|metacaspase	7			7
iTMHo|metacaspase	6	1		7
iTMHo|metacaspase|VWA_2		5 (5)		5 (5)
Metacaspase|FGE-sulfatase		2 (2)	3 (3)	5 (5)
Metacaspase|CarboxypepD_reg	1			1
Metacaspase|metacaspase			1	1
Metacaspase|PBP_like_2			1 (1)	1 (1)
Metacaspase|YEATS			1	1
MORN|metacaspase	2		1	3
MORN × 2|metacaspase	1			1
MORN × 3|metacaspase	1			1
MORN × 4|metacaspase		1		1
Sel1 × 3|metacaspase			1	1

Values in parentheses are cyanobacterial metacaspases. Domains are in order from N-terminus to C-terminus and are separated by “|.” Transmembrane helices are indicated by iTMHo or oTMHi, where “i” and “o” correspond to inside and outside the cell, respectively. “Peptidase_C14” is the Pfam caspase domain, which in some cases is a better match than the bacteria-specific metacaspase domain. Repeated domains are indicated by “ × n,” where n is the number of repeats.

**Figure 3:**
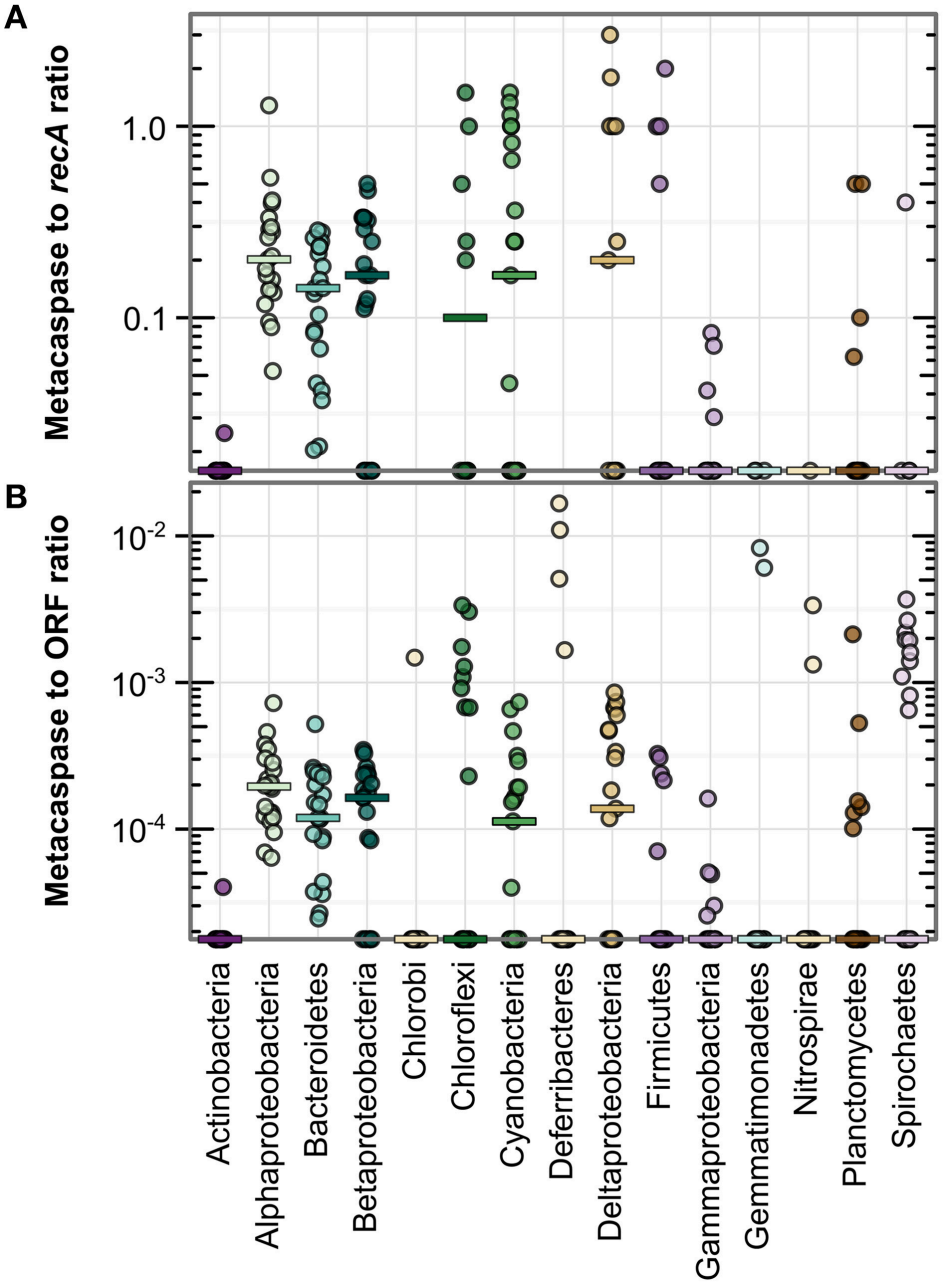
**Metacaspase gene abundance within bacterial groups along the Baltic Sea transect (Phase I; 21 stations)**. Abundance is given as the ratio of bacterial metacaspase to *recA* sequences **(A)** or the ratio of bacterial metacaspase sequences to all bacterial ORFs **(B)**. Values are presented on a log-10 scale, with zeros at the bottom of each panel. The horizontal bars indicate medians. Significant differences are given in Table S3. Note the absence of data points for Chlorobi and Deferribacteres in **(A)** due to the lack of detection of *recA* sequences in these groups.

About 4% of Baltic Sea bacteria represented in the Phase I transect metagenomes carried metacaspase genes (as estimated using *recA* abundances; see Materials and Methods). However, as seen in Figure 2B, the relative taxonomic affiliation and distribution patterns of *recA* and the metacaspase genes differed markedly. The metacaspase genes were primarily affiliated to Alpha- and Betaproteobacteria, and to some extent to Deltaproteobacteria, in the low salinity northern waters, while their hosts were substituted by Bacteroidetes and Cyanobacteria in the central Baltic Sea. In the marine environments, metacaspase diversity was dominated by Alphaproteobacteria. In contrast to other groups, the distribution of betaproteobacterial metacaspase genes along the transect (decreasing; Figure 2B) is positively correlated (Pearson's *r* ≈ 0.88) with the betaproteobacterial *recA* count distribution (decreasing; Figure 2A). Notably, despite representing a large proportion of the bacterial community, metacaspase genes were rare in Actinobacteria and Gammaproteobacteria and were not found in Verrucomicrobia (Figures 2B, 3) nor unicellular Cyanobacteria (Table S1).

Figure 3 further illustrates the elevated metacaspase abundance in Alphaproteobacteria, Bacteroidetes, Betaproteobacteria, Cyanobacteria, and Deltaproteobacteria. These groups showed a median metacaspase gene abundance of 0.1–0.2 per *recA* gene (Figure 3A) or 0.1–0.2%0 of all ORFs (Figure 3B). The former value can be interpreted as presence of metacaspases in at most 20% of the genomes, as *recA* is expected to be represented by one copy per bacterial genome. In a subset of the samples, an abundance of one metacaspase sequence per genome or higher was observed in Alphaproteobacteria, Chloroflexi, Cyanobacteria, Deltaproteobacteria, and Firmicutes (Figure 3A). A high ratio vs. *recA* could however simply be directly related to the genome size, with greater numbers of metacaspases in larger genomes. It may therefore also be relevant to consider the ratio between metacaspase genes and the total number of ORFs, as it indicates how much of the genome the organism devotes to this particular function. Comparing normalization to *recA* (Figure 3A) and to ORFs (Figure 3B), it is apparent that the general patterns distinguishing groups remain intact, with a few exceptions. For example, Deltaproteobacteria and Firmicutes appeared to show a comparatively lower abundance per ORF than per genome (*recA*), while the opposite is true for Chloroflexi in a few samples.

Importantly, a high proportion (one to two fifths) of the detected bacterial metacaspase sequences are of unknown bacterial origin (Figure 2B), a considerably higher proportion than that observed for *recA* (Figure 2A). If these sequences are affiliated with any of the groups mentioned above it is possible that their metacaspase abundances have been underestimated.

By evaluating APIS taxonomic classifications at the genus level it became apparent that some bacteria were particularly rich in metacaspase genes (Table S2). In fact, the 20 most metacaspase-rich genera (3% of all genera) held 37% of all bacterial metacaspases. More than two genes per every 1000 genes (2%0) encoded metacaspases in the bacteria *Candidatus* Accumulibacter, *Roseburia, Magnetospirillum, Denitrovibrio, Mesorhizobium, Labrenzia, Beggiatoa, Xanthobacter, Leeuwenhoekiella*, and *Nostoc*, constituting the top 10 genera. This value (2%0) is high compared to the medians observed in the different bacterial groups (0–0.2%0of all ORFs; Figure 3B).

The metacaspase sequences identified in the Phase I metagenomes are presented in Table S1 and Data Sheet S3.

#### Comparison of metacaspase gene abundance in different size fractions

**Figure 4:**
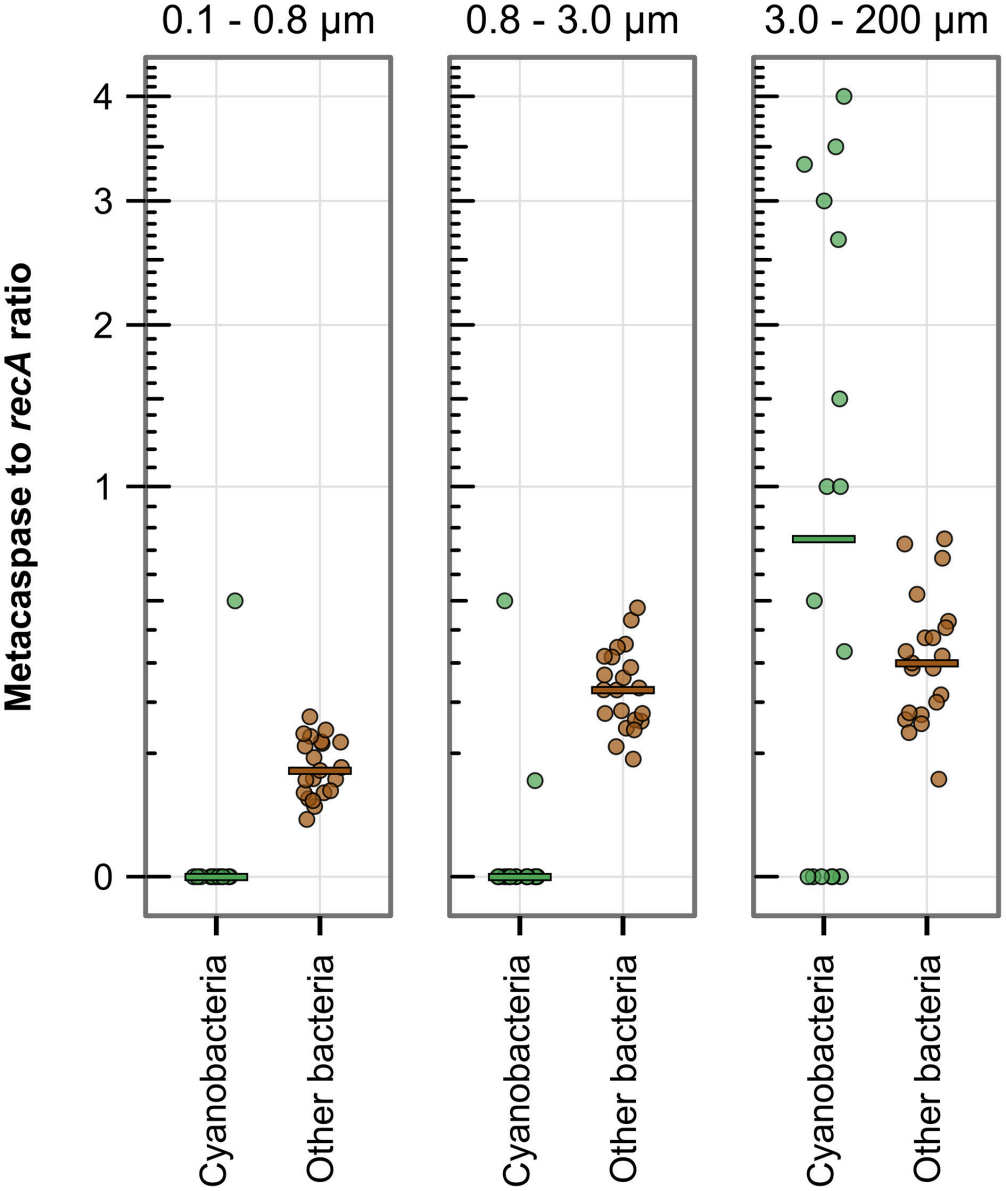
**Metacaspase gene abundance among bacteria compared by size fraction in the metagenomes along the 2009 Baltic Sea transect (Phase I; 21 stations)**. The abundance is the ratio of bacterial metacaspase to *recA* sequences, displayed on a square root scale. The horizontal bars indicate the median values. When combining Cyanobacteria and Other bacteria, the two larger size fractions are significantly different from the smaller, i.e., the larger size fraction bacteria have a higher metacaspase abundance. Significant differences are given in detail in Table S3.

Examining the bacterial genomic abundance of metacaspase sequences after size fractionation of the microbial populations revealed a possible positive correlation to size (Figure 4). The ratio of metacaspase genes to *recA* in the smallest fraction (0.1–0.8 μm) was on average 0.083 (0.11%0 of all bacterial ORFs) while 0.23 (0.19%0) in 0.8–3.0 μm and 0.44 (0.34%0) in the 3.0–200 μm size fraction. The abundance in the larger fractions was significantly higher than that in the smallest fraction, using normalization to ORF as well as *recA* counts (Table S3), suggesting that the correlation to size is not only an effect of larger genome sizes. Metacaspase abundance in the larger Cyanobacteria of the 3.0–200 μm size fraction was not significantly higher than in other bacteria, yet a few samples showed several fold higher abundance levels for Cyanobacteria (Figure 4).

### Analysis of temporal genetic data (Phase II metagenomes)

The second stage of the survey focused on seasonal microbial patterns in the Baltic Proper. The sampling performed off the Stockholm archipelago island Askö (see Figure 1) yielded a metagenomic dataset consisting of six size fractionated samples (3.0–200, 0.8–3.0, 0.1–0.8 μm, and a viral fraction, for a total of 6 × 4 = 24 metagenomes) collected from June to August in 2011 as previously described in Larsson et al. (2014). These Phase II metagenomes consist of a total of 36,489,579 ORFs. As with the Phase I transect metagenomes, results (gene hits) derived from the viral samples are only included in Table S1. Sequencing data is available via NCBI BioProject PRJNA322246.

#### Bacterial summer community composition

**Figure 5:**
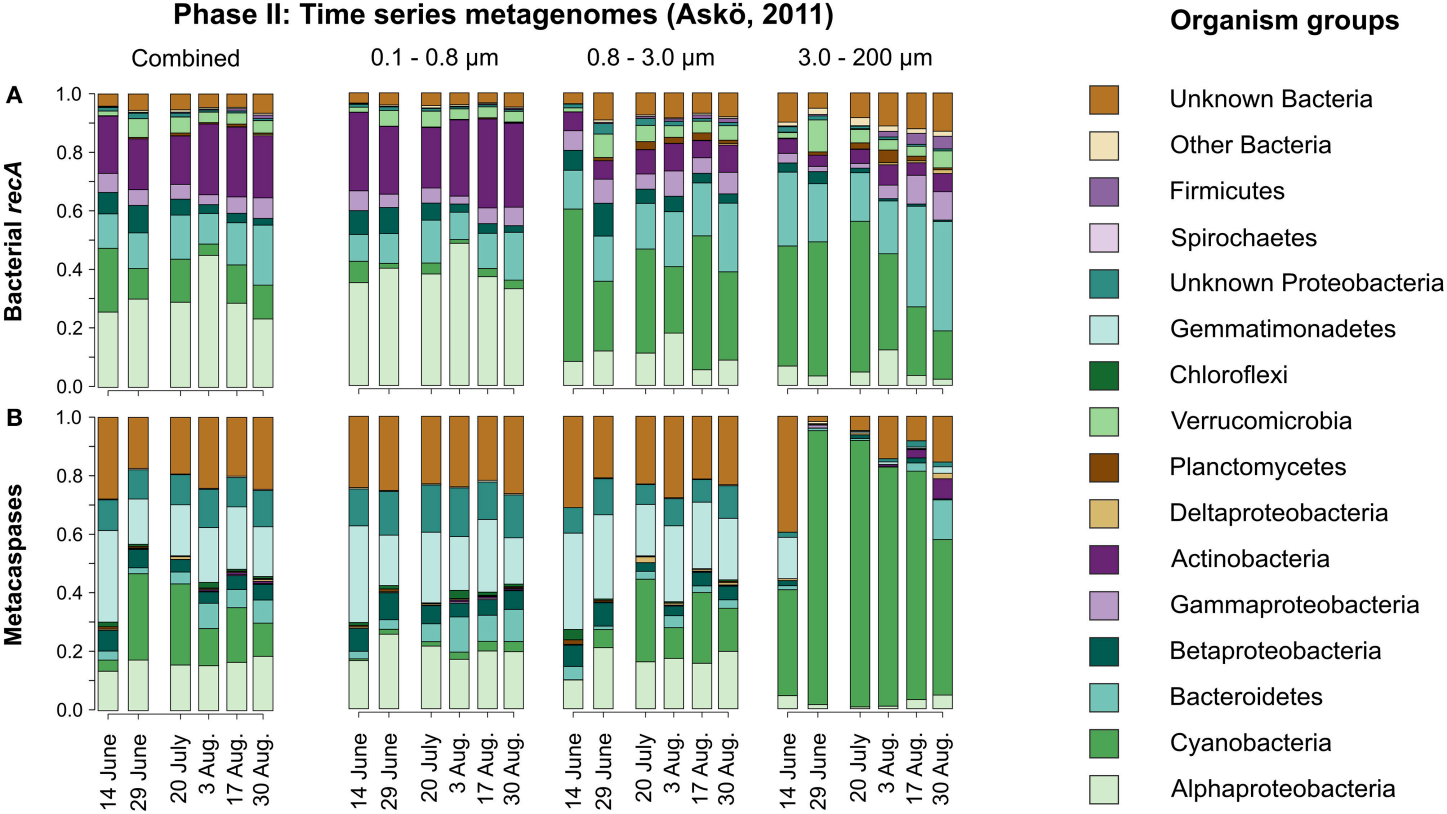
**Taxonomic distribution of bacterial ***recA*** (A) and metacaspase genes (B) in metagenomes derived from the time series of the summer season 2011 at Askö (Phase II; 6 time points, June–August)**. The relative values are summed average nucleotide coverages for the assembled contigs to which the identified genes belong. The data are presented for each of the non-viral size fractions individually as well as combined by date.

Figure 5A illustrates the seasonal variation in taxonomic affiliation of bacteria based on the *recA* gene. Members of Alpha- and Gammaproteobacteria as well as Bacteroidetes, Actinobacteria, and to some extent Betaproteobacteria and Verrucomicrobia contributed significantly to the temporally rather stable Phase II bacterial summer community. Notably, a mix of Cyanobacteria dominated the two larger size fractions through the sampling season (Figure 5A), with the unicellular order Chroococcales being most abundant (Figure S4). Larger celled and filamentous Cyanobacteria belonging to the orders Nostocales and Oscillatoriales were particularly common in the 3.0–200 μm size fraction (35% of all cyanobacterial ORFs), while rare in the 0.8–3.0 μm size fraction (3%).

The *recA* sequences identified in the Phase II metagenomes are presented in Table S1 and Data Sheet S3.

#### Seasonal taxonomic composition of metacaspase genes

As seen in Figure 5B, bacterial metacaspase genes of diverse taxonomic origin were detected at all time points sampled in the summer time series (Phase II). In total, 370 metacaspase sequences were identified over the season (Table 1).

The metagenomes yielded an average per-sample metacaspase to *recA* coverage ratio of 0.08, suggesting that at most 8% of the sampled bacterial genomes may hold metacaspases. Cyanobacterial metacaspase genes dominated the large size fraction (Figure 5B), surpassing the abundance of Cyanobacteria (compare Figures 5A,B). The metacaspase genes were frequently affiliated with Alphaproteobacteria, but also showed frequent association with Gemmatimonadetes, over the summer in the smaller size fractions (0.1–0.8 and 0.8–3.0 μm). Despite the absence of *recA* affiliated with Gemmatimonadetes in our dataset (Figure 5A), *recA* is present in this phylum (see e.g., UniProt entry W0RP79 in Data Sheet S2). Conversely, metacaspase genes were underrepresented among Actinobacteria, Gammaproteobacteria, and Verrucomicrobia (Figure 5B). Furthermore, only one metacaspase gene was retrieved from a unicellular cyanobacterium (*Microcystis*; Table S1), which is in contrast to the high abundance of the unicellular Chroococcales during the season (Figure S4).

As with the Phase I transect metagenomes, a large proportion (ca. 20–25%) of the bacterial metacaspase genes could not be classified taxonomically better than to the Bacteria domain level (Figure 5B).

The metacaspase sequences identified in the Phase II metagenomes are presented in Table S1 and Data Sheet S3.

#### Genomic contexts of bacterial metacaspase genes

In order to obtain more detailed information about the role of metacaspases in natural microbial populations, the genomic context of metacaspase genes was examined. Seven unique contig variants with bacterial metacaspase genes and at least two additional adjacent genes were identified in the Phase II metagenomic dataset (Figure S5). Three of the metacaspase genes on the contigs are classified as cyanobacterial, whereas the other four are belonged to Proteobacteria, Bacteroidetes, Gemmatimonadetes, and Actinobacteria. It may be noted that on three of the contig variants the metacaspase genes are in close vicinity to genes involved in sulfur metabolism (see also below).

### Analysis of gene expression data (Phase I and Phase III metatranscriptomes)

Entering its third stage, the survey shifted focus from the genetic background to the microbial metacaspase gene expression and thereby potential activity of metacaspases in the Baltic Sea. Analysis of the spatially (Phase I; transect) and temporally (Phase III; time series) sampled and co-assembled metatranscriptomes uncovered the expression of 64 unique metacaspase genes, out of which 54 were of bacterial origin (Table 1). The remainder was eukaryotic (five sequences) or affiliated with unknown cellular organisms (five sequences). All 64 expressed metacaspase genes are listed as M1 to M64 in Table S1, and the corresponding amino acid sequences are provided in Data Sheet S3. Sequencing data is available via NCBI BioProject PRJNA320636.

**Figure 6:**
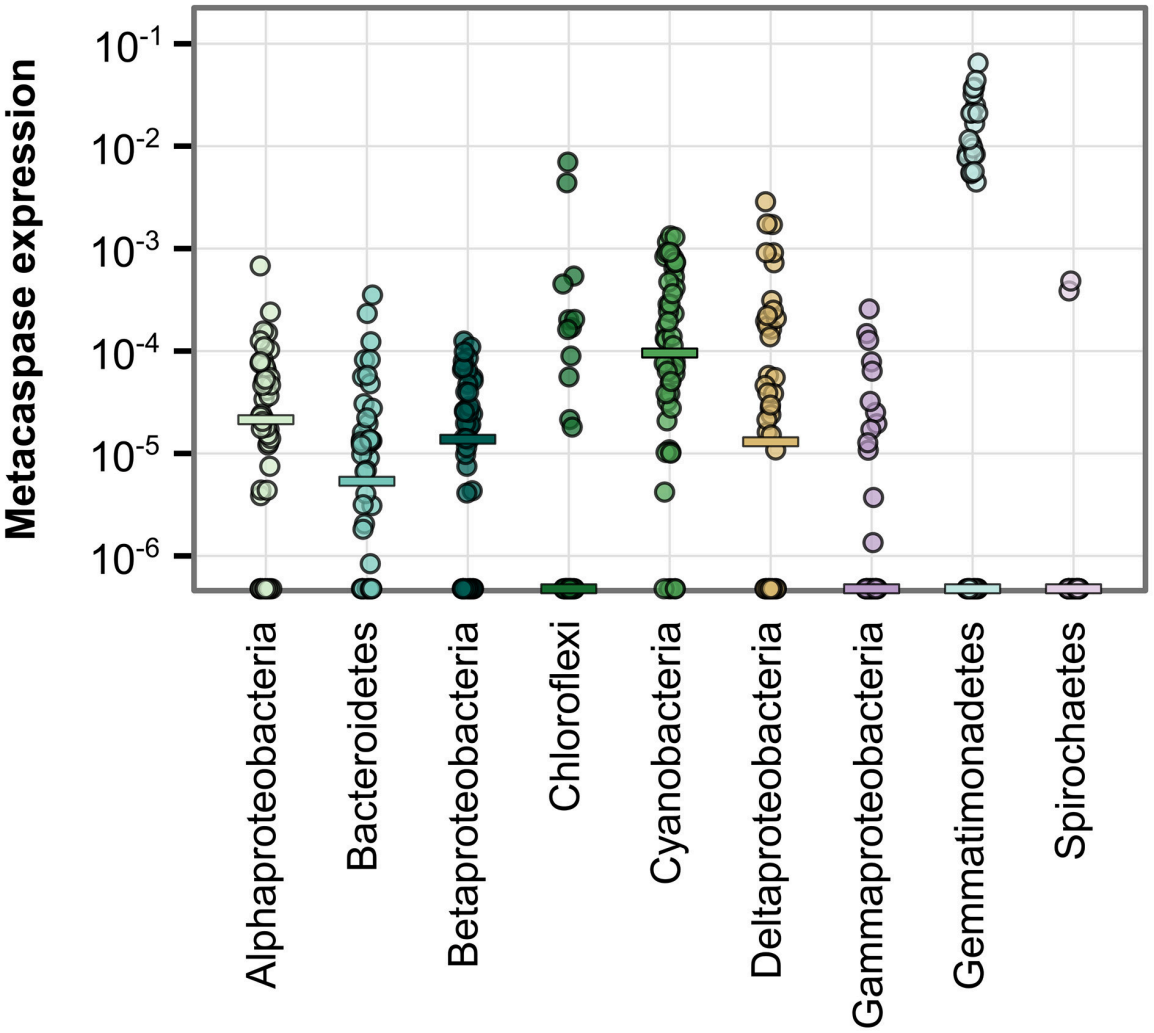
**Metacaspase gene expression level by bacterial group**. Each point represents one metatranscriptomic sample (Phase I and III; 50 samples). Expression levels are given as the number of reads mapping to bacterial metacaspase transcripts divided by the total number of reads mapping to bacterial transcripts. Values are presented on a log-10 scale, with zeros at the bottom of each panel. Horizontal bars indicate medians. Significant differences are described in Table S3.

The cyanobacterial phylum showed the highest number of transcribed metacaspase genes (20; Table 1). The metacaspases classified at genus level were partitioned into *Anabaena* (10 genes), *Nodularia* (three genes), *Nostoc, Calothrix, Crinalium, Cyanothece*, and *Microcystis* (one gene each). In addition to the high median metacaspase gene expression in Cyanobacteria (Figure 6), expression was also detected among Bacteroidetes, Chloroflexi, Gemmatimonadetes, and Spirochaetes as well as among Proteobacteria, although these showed fewer unique metacaspase transcripts. The metacaspase gene expression level was commonly between 10 and 100 ppm within groups, based on metacaspase read counts divided by total read counts in the group. Although other groups rivaled the expression level of Cyanobacteria in many samples, the overall magnitude (10 ppm—1%0) is still significantly higher in Cyanobacteria compared to other groups (Table S3). The exception is Gemmatimonadetes, which is not significantly different.

Metacaspase gene expression levels were not significantly different between size fractions either for bacteria in general or for non-cyanobacterial bacteria. However, Cyanobacteria in the largest size fraction (3.0–200 μm) showed a significantly higher metacaspase expression level compared to non-cyanobacteria in the 0.1–0.8 μm fraction (Table S3).

**Figure 7:**
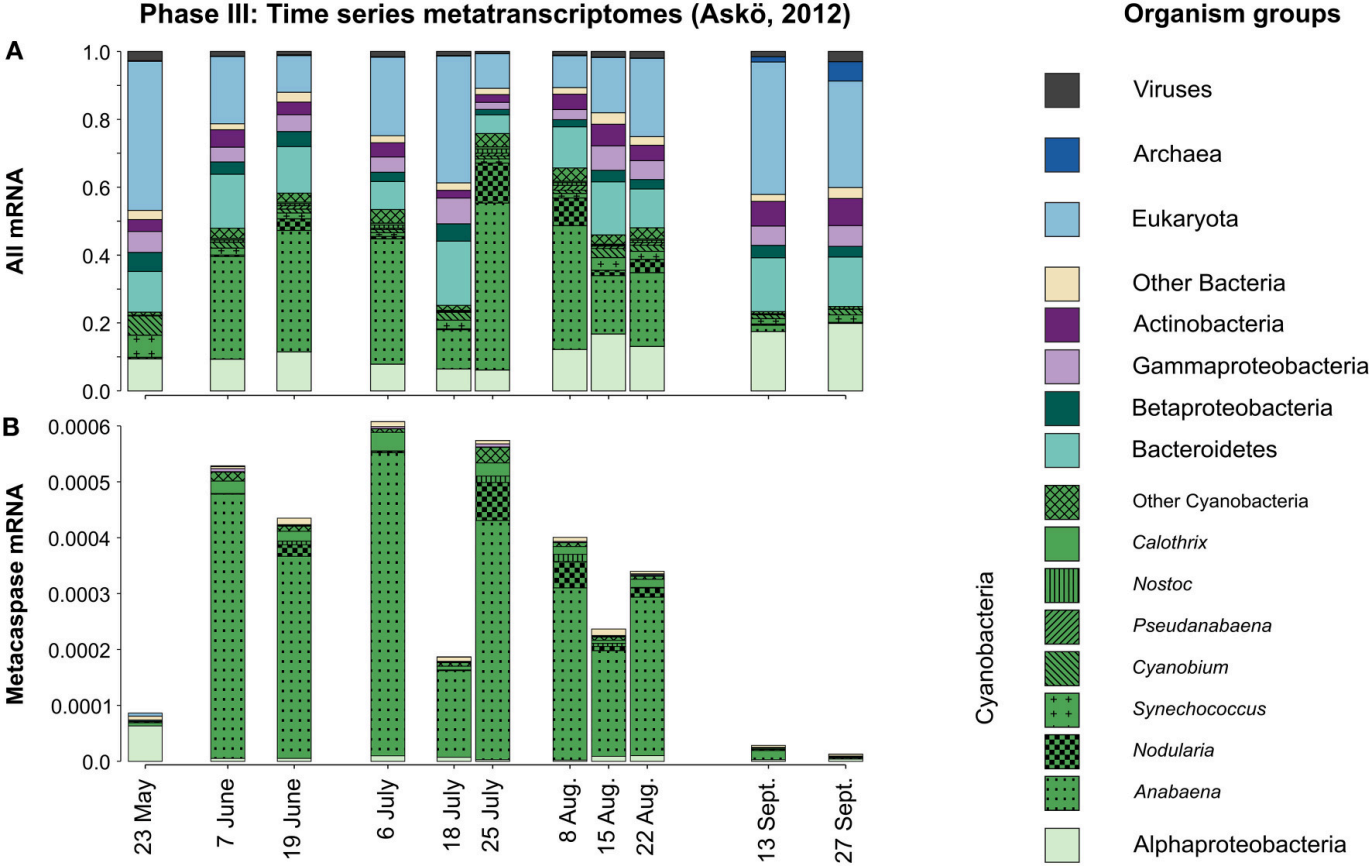
**Taxonomic distribution of all microbial groups (A) and their expressed metacaspase genes (B) in the metatranscriptomes sampled during the 2012 Askö time series (Phase III; 11 samples, May–September)**. The displayed values are based on mapped read counts. For metacaspase genes the read counts were divided by the grand total number of mapped reads, thus showing the expression of these genes relative to the whole community transcription **(B)**. Note that Cyanobacteria are presented at the genus level.

Examining the Phase III seasonal metatranscriptomes (May to September 2012) separately (Figure 7A), it is apparent that these were mostly dominated by Cyanobacteria, but with notable contribution by Alpha-, Beta-, and Gammaproteobacteria as well as Bacteroidetes and Actinobacteria. On average 60% (peaking at 87%) of the cyanobacterial mRNA could be traced to the filamentous genera *Anabaena* and *Nodularia*. *Anabaena* is probably to a large extent representing *Aphanizomenon* sp., a close relative (Janson and Granéli, 2002; Rajaniemi et al., 2005) that lacks a Baltic Sea reference genome. Including all cellular transcripts, as opposed to investigating the *recA* gene in the metagenomes, reveals that a significant portion of the community mRNA is of eukaryotic origin (Figure 7A).

Screening the corresponding metacaspase gene transcription over the season (Phase III time series) it was immediately apparent that the expression of these genes was almost exclusively dominated by filamentous Cyanobacteria (Figure 7B). Moreover, the transcript abundance followed the rises and falls of the cyanobacterial populations (blooms) from early June to the end of August (Figure 7A).

### Metacaspase expression in a *Nodularia* bloom: A natural experiment

In the fourth and final stage of analysis we sought to study metacaspase gene expression in a more focused and detailed context, aiming at identifying possible functional roles of metacaspases. As previously shown, expression of specific genes may differ significantly under laboratory and field conditions, respectively (Price et al., 2014). This dataset therefore constitutes a unique opportunity to explore the influence of natural environmental parameters on metacaspase gene expression patterns.

After discovering the dominance of Cyanobacteria in metacaspase gene expression we singled out one of the major bloom-forming species for a natural *in situ* gene expression “experiment” set against the cyanobacterial bloom backdrop (see Figure S2 for population monitoring data). Initial screening of the Phase III seasonal metatranscriptomic time series suggested that the cyanobacterium *Nodularia spumigena* would be a suitable subject as it yielded read counts of up to 1.3 million per sample (Figure S1). Out of the 2417 genes passing filtration, 95% had ≥ 10 mapped reads in at least 50% of the samples, and 5% had 10 or more mapped reads in all samples (Figure S6). Further, blastn mapped 96.2% of the transcripts classified as *N. spumigena* in our metatranscriptome to the sequenced genome (*N. spumigena* CCY9414; Voß et al., 2013), and 87.5% of the transcripts aligned at more than 98% identity (Figure S7).

**Figure 8:**
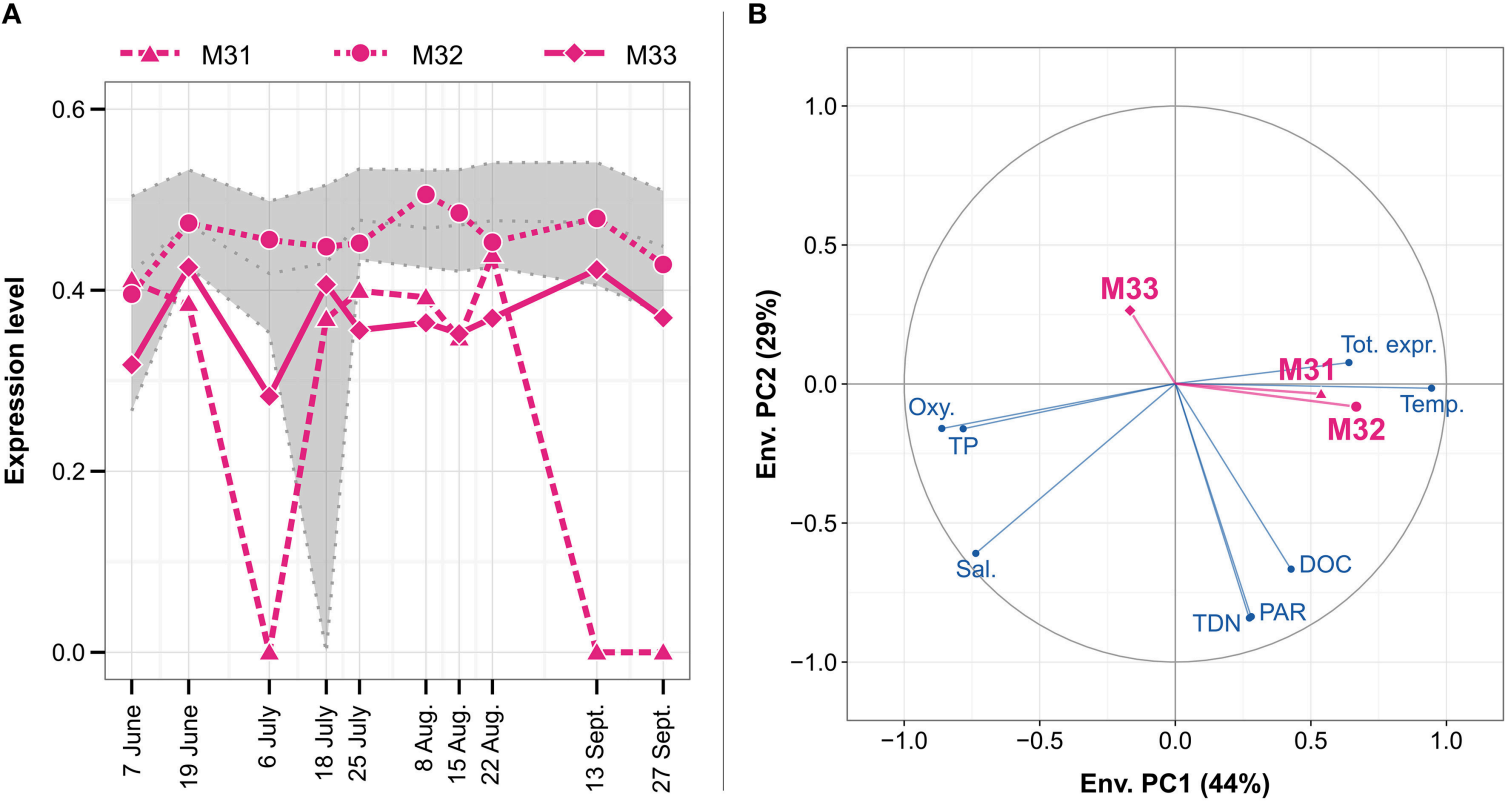
**Expression patterns for the three ***Nodularia spumigena*** metacaspase genes M31–M33 (A) and their correlation to environmental variables (B) during the Askö 2012 time series (Phase III; 10 samples, June–September)**. The expression level **(A)** is given in inverse hyperbolic sine transformed RPKM values divided by the maximum in each sample. The gray dotted lines represent, from top to bottom, the upper quartile, median and lower quartile of the expression level of *N. spumigena* genes. The circle of correlations **(B)** shows Pearson correlation coefficients between the environmental variables (blue) or metacaspase expression patterns (magenta) and the first two principal components of the scaled and PCA-transformed environmental data (see also Figure S2). The proportion of the variance that is explained by each principal component is shown in parentheses in the axis titles. The environmental variables are water temperature (Temp.), oxygen (Oxy.), salinity (Sal.), dissolved organic carbon (DOC), total dissolved nitrogen (TDN), total phosphate (TP), photosynthetically active radiation (PAR), and total expression of *N. spumigena* relative to all mRNA (Tot. expr.).

The genome of *N. spumigena* encodes for three metacaspase genes termed NSP_13290, NSP_23610, and NSP_48590, corresponding to metacaspases M31, M32, and M33, respectively, in the Baltic Sea metatranscriptome (Figure 8A). Whereas M32 showed a fairly constant expression level close to the median, M33 and M31 were more divergent, generally having a lower expression level than M32. M31 displayed the most varying expression pattern with peaks early in June and later in July and August. The small number of observation time points (10) prevented certain statistical analyses (e.g., correlation testing and multiple linear regression) and no distinct environmental variables explaining the seasonal metacaspase expression patterns could be identified. However, correlation data suggested that M33 responded to different environmental variables, such as reduced sunlight, total nitrogen, and organic carbon concentration, compared to M31 and M32, which may follow temperature and population size to some extent (Figure 8B).

**Figure 9:**
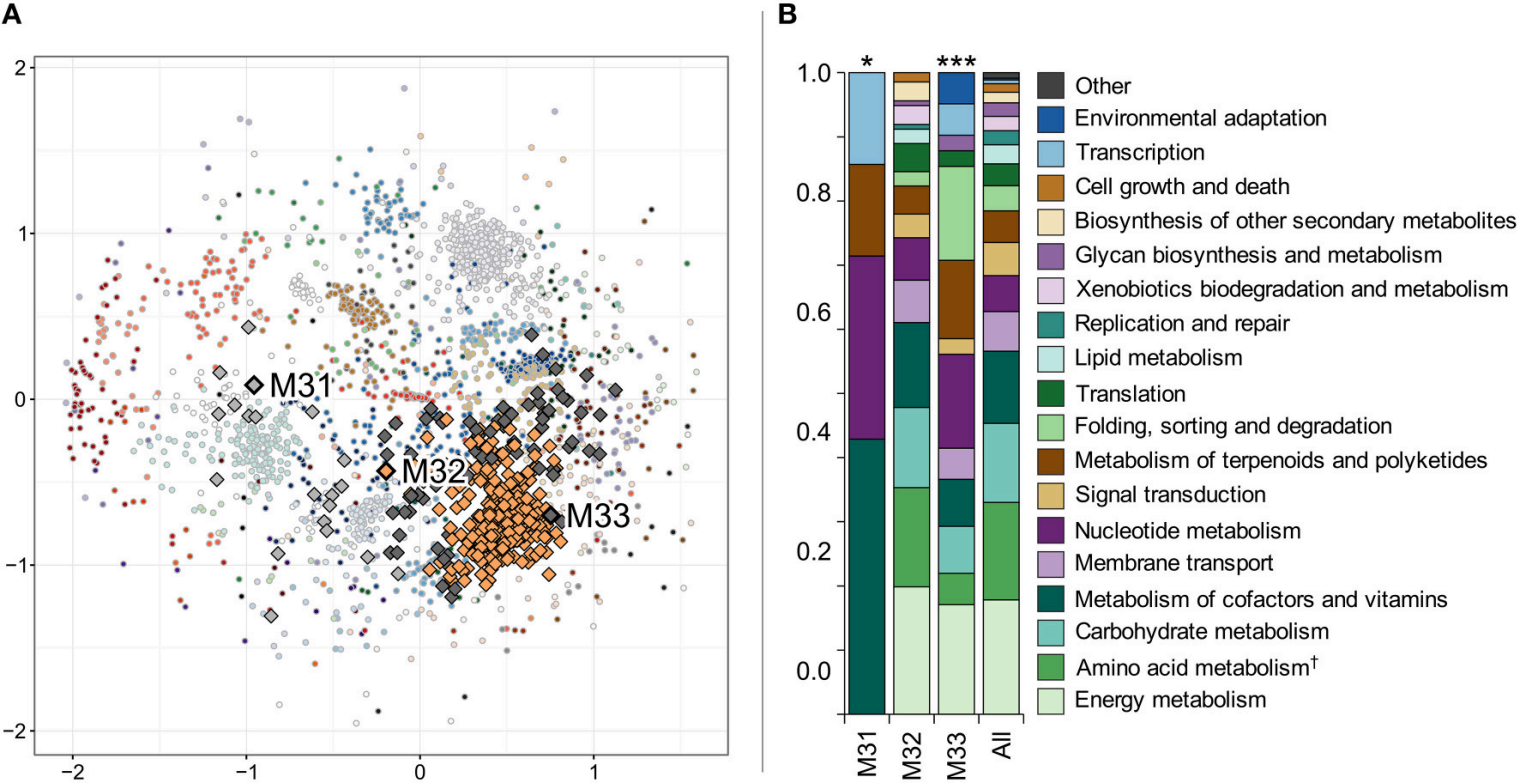
**Hierarchical clustering of ***Nodularia spumigena*** gene expression patterns based on Pearson correlation distance (A) and functional classification of the three clusters containing metacaspases M31–M33 (B)**. Each dot in **(A)** represents one gene and each color represents one of the 62 clusters. The genes have been placed according to their non-metric multidimensional scaling coordinates in two dimensions based on the Pearson correlation distance matrix. The three metacaspases M31 to M33 and their clusters (defined by a hierarchical clustering tree cut into clusters to yield the highest average silhouette width; see Materials and Methods) have been emphasized with a black outline and larger markers. **(B)** shows gene functions of the metacaspase-containing clusters based on KEGG ortholog annotations. A chi-square test was performed in order to determine whether the distribution of functions was significantly different in the metacaspase clusters compared to all expressed genes in *Nodularia spumigena*. The results are shown above the bars representing each cluster (^*^p < 0.05 and ^***^p < 0.001). “Amino acid metabolism^†^” is the combination of the categories “Amino acid metabolism” and “Metabolism of other amino acids.” The “Other” functional category consists of “Cell motility,” “Drug resistance,” and “Transport and catabolism.”

The 2417 genes constituting the *N. spumigena in situ* transcriptome were grouped into 62 clusters based on their expression patterns (average silhouette width 0.36). M31, M32, and M33 were separated into three different clusters consisting of 20, 278, and 78 genes, respectively (Figure 9A, Table S4). KEGG functional annotations showed that the cluster compositions of M31 and M33 (Figure 9B) differed significantly from the union of all clusters (chi-square test with *p* = 0.033, 0.00030, respectively).

The metacaspase genes M31 and M33 occupied gene expression clusters that also include three transcript ORFs annotated as KEGG orthologs K16128, K16129, and K16134 (Table S4), belonging to the category “Metabolism of terpenoids and polyketides.” Blastn vs. the nodularin gene cluster from *N. spumigena* NSOR10 (GenBank accession AY210783.2) yielded close to perfect matches (full-length alignments of >98% identity), confirming that these ORFs are nodularin synthesis genes.

The M33 expression cluster also included a sigma-B regulatory phosphatase RsbU (NSP_8730) and showed enrichment of the functional categories “Folding, sorting, and degradation” and “Environmental adaptation” (Figure 9B). Furthermore, the M33 expression cluster encompassed the heterocyst-related genes *hetR* (NSP_16830) and heterocyst glycolipid synthase (NSP_46340). The latter (NSP_46340) was also found in the M32 gene expression cluster, but mapping to a different metatranscriptomic ORF (possible explanations for such observations is natural strain microdiversity or the presence of incomplete ORFs after assembly). NSP_32600 (encoding an ABC-transporter component) is yet another heterocyst-associated gene that appeared in the M32 expression cluster.

**Figure 10:**
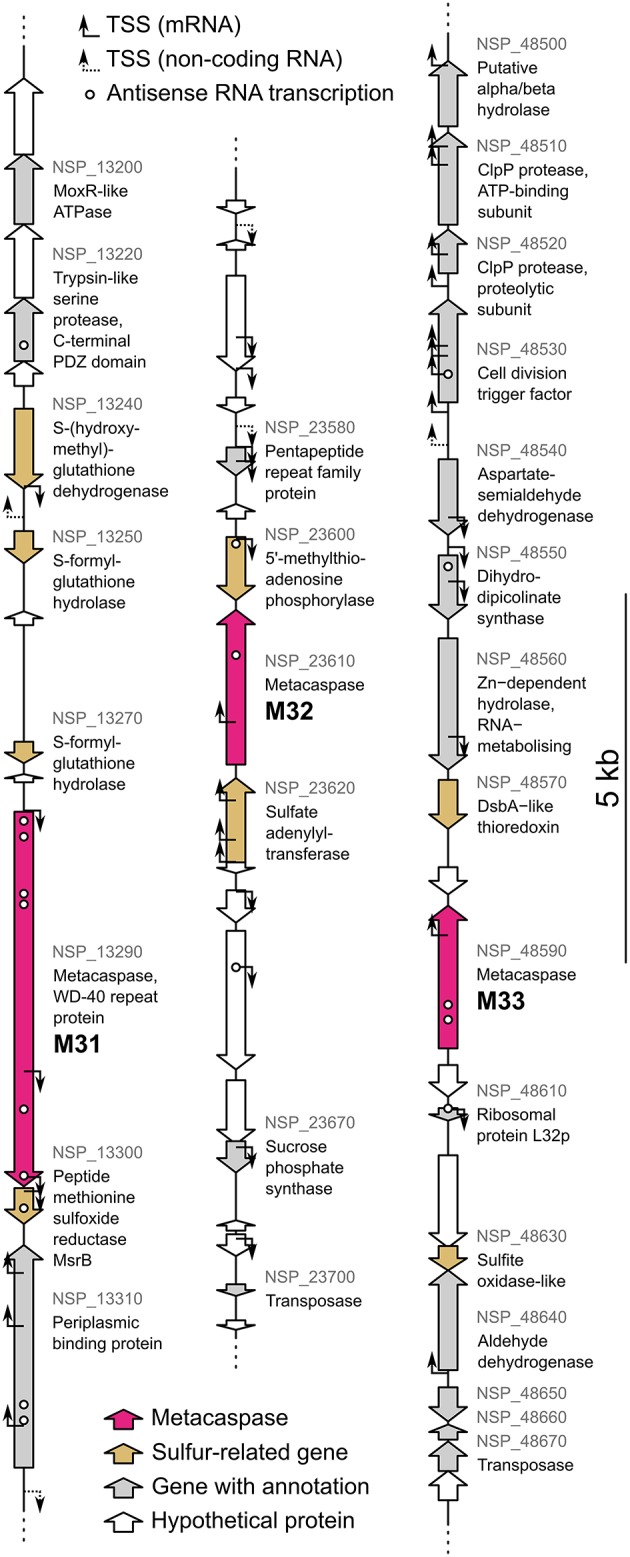
**The ***Nodularia spumigena*** CCY9414 genome loci of the metacaspases M31 (NSP_13290), M32 (NSP_23610), and M33 (NSP_48590)**. Gene annotations are based on GenBank accession CP007203.2 (Voß et al., 2013). Locus tags are shown in gray for genes with an annotation. The original annotations identify M31 as a WD-40 repeat protein and M32 and M33 as hypothetical proteins. Transcription start sites (TSS) for mRNA and non-coding RNA as well as expressed antisense RNAs previously identified by Kopf et al. (2015) are indicated.

Within the sequenced *N. spumigena* CCY9414 genome, all three metacaspase genes are co-localized with two or more genes related to sulfur metabolism (Figure 10). These encode S-(hydroxymethyl)-glutathione dehydrogenase, S-formyl-glutathione hydrolase and peptide methionine sulfoxide reductse MsrB at the M31 locus, 5′-methylthioadenosine phosphorylase and sulfate adenylyltransferase at the M32 locus and DsbA-like thioredoxin and a sulfite oxidase-like protein at the M33 locus. Two genes belonged to the same gene expression cluster as their neighboring metacaspase gene, i.e., metacaspase M33 (Table S4). These co-localized and co-expressed genes encode an RNA-metabolizing Zn-dependent hydrolase (NSP_48560) and a hypothetical protein (NSP_48580), neither demonstrating any obvious sulfur connections.

## Discussion

The present work constitutes the first study on community-wide abundances of genes encoding the metacaspase protease in a natural aquatic microbial community. This was achieved through analyses of comprehensive metagenomic and metatranscriptomic datasets from microbes inhabiting a temperate brackish water ecosystem, the Baltic Sea, and surrounding waters. The microbial populations were examined spatially (through a steep salinity gradient; 0–35 PSU) and temporally, at a site supporting cyanobacterial blooms, over two summer seasons. Not only the metacaspase gene abundance and taxonomic affiliations, but also metacaspase gene expression patterns were examined. The transcription level approach allowed a general assessment of the expression of these genes, and is more appropriate than measuring proteolytic activity considering the poorly characterized, and possibly diverse, target specificity of the encoded proteins.

### Metacaspases represent a specialized adaptation

A small subset of Baltic Sea bacteria (4%) was found to harbor genes encoding metacaspases. This frequency is considerably lower than that recently reported (19%) from screening 1349 sequenced bacterial genomes (Asplund-Samuelsson et al., 2012). However, similar to the observation made among sequenced genomes, metacaspases in the Baltic Sea are found in multiple bacterial phyla (Figure 3). Simultaneously, a large proportion of the metacaspase gene sequences only allowed classification at the Bacteria domain level (Figures 2B, 5B), suggesting that some naturally occurring bacterial metacaspase hosts belong to taxonomic groups still lacking appropriate reference genomes, for example from the metacaspase-deprived Actinobacteria, Gammaproteobacteria, and Verrucomicrobia, as also identified previously (Asplund-Samuelsson et al., 2012). Note that the higher propensity for correct estimates when annotating the more conserved gene *recA* (Figure 2A) compared to metacaspase sequences (Figure 2B) may reduce the estimated percentage of bacteria carrying metacaspase genes. These observations still suggest that metacaspases constitute specialized adaptations connected to a specific physiology or lifestyle of the bacteria carrying them, rather than a general bacterial function.

The Baltic Sea is characterized by gradients in salinity and nutrient concentrations. Thus, the distribution of organisms and genetically encoded functions along these gradients may point to specific adaptive traits. While no clear geographical pattern in the abundance of the metacaspase genes along the Baltic Sea transect was apparent (Figure S3), certain taxonomic affiliation patterns were detectable. For instance, the high incidence of Bacteroidetes metacaspase genes in the phytoplankton-rich central parts of the transect (Dupont et al., 2014), suggest that metacaspases may be involved in the degradation of phytoplankton derived polymeric organic compounds known to be carried out by these bacteria (Thomas et al., 2011). Furthermore, as the proportion of Betaproteobacteria carrying metacaspases was constant over the transect (i.e., correlated with *recA*; Figure 2), and higher than in sequenced betaproteobacterial genomes (Asplund-Samuelsson et al., 2012), metacaspases may constitute an adaptation of particular importance to this bacterial group. Betaproteobacteria have been shown to influence nutrient cycling in freshwater ecosystems (Parveen et al., 2011) and it could therefore be speculated that these bacteria also employ metacaspase proteases for extracellular protein degradation, rather than cellular differentiation or multicellular behavior including PCD.

Size fractionation by serial filtration is a widespread technique in aquatic microbiology (Rusch et al., 2007; Karsenti et al., 2011; Wrighton et al., 2012). However, this technique is not without flaws, specifically smaller bacteria can get caught on larger filters, similar to a logjam in a river (Sheldon and Sutcliffe, 1969; Padilla et al., 2015). Differences in taxonomy, functional gene content, and genome size according to filter fraction have been observed consistently across multiple studies by several research groups (see e.g., Zeigler-Allen et al., 2012; Crespo et al., 2013; Ganesh et al., 2014; Simon et al., 2014). If unintended retention of free-living bacteria on the larger size fraction filters does indeed occur, this would result in a numerical and genomic dilution of the true particle-attached bacteria. Therefore, we believe that the differences detected between size fractions in this and other studies are not only statistically robust when shown, but likely conservative measurements. The higher metacaspase gene abundance observed in the two larger size fractions suggests that larger, particle-associated bacteria, or bacteria with surface properties that make them retain on the larger filters, contain on average a higher number of metacaspases. Particle-associated bacteria experience a microenvironment that is distinct from that inhabited by free-living bacteria (Simon et al., 2014), characterized by increased substrate availability as well as complex interaction between organisms (Grossart et al., 2003). In this context, metacaspase proteases could facilitate substrate degradation or fine-tuning of population dynamics through PCD. Bacteria in the Baltic Sea with higher number of metacaspase genes also include bacteria with multicellular lifestyles, such as the filamentous gammaproteobacterium *Beggiatoa* as well as filamentous Chloroflexi (Overmann, 2008; Salman et al., 2013).

One striking feature of our datasets is that among all the identified microorganisms in the Baltic Sea filamentous representatives from the photoautotrophic phylum Cyanobacteria ranked among the bacteria that both showed the highest metacaspase abundance and highest metacaspase gene expression levels. These Section IV filamentous Cyanobacteria (Rippka et al., 1979) are characterized by an advanced cell differentiation machinery (vegetative cells differentiate into akinetes, hormogonia, and nitrogen fixing heterocysts), the biosynthesis of a multitude of secondary metabolites including potent toxin, and their conspicuous bloom formations. We hypothesize that these cyanobacteria or other large or particle-associated bacteria (Figure 4), with larger and less streamlined genomes (Dupont et al., 2014), possess a wider repertoire of metacaspases that may be functionally diversified as to benefit a complex lifestyle.

### *Nodularia spumigena* metacaspase genes are functionally diversified

The three metacaspase gene variants (M31–M33) identified here in the Baltic Sea bloom-forming *Nodularia spumigena* and their expression patterns further stress the functional diversification of cyanobacterial metacaspases. For instance, M31 seems to have a specialized function, as it clusters with only a handful of other genes. In contrast, the placement of the *N. spumigena* M32 metacaspase gene in a functionally generalized gene expression cluster and an expression level close to the median (Figure 8A), suggest house-keeping functions of this gene. For M33, finally, the results suggest a stress-related function as the gene responds differently to environmental variables compared to both M31 and M32 (Figure 8B), it correlates with the known stress response gene RsbU (Table S4; Shin et al., 2010), and among genes that it clusters with, the categories “Folding, sorting, and degradation” and “Environmental adaptation” are enriched (Figure 9B).

Another function of M33 connected to stress may be the potential involvement of this gene in cyanobacterial cell differentiation, specifically heterocyst formation. Both the heterocyst master regulatory gene *hetR* (Kumar et al., 2010) and a heterocyst glycolipid synthase gene (NSP_16830 and NSP_46340; Table S4) belong to the same gene expression cluster as M33. Indeed, heterocysts define Section IV filamentous nitrogen-fixing cyanobacteria (Rippka et al., 1979), to which the Baltic Sea dominating bloom-forming cyanobacteria *Nodularia, Aphanizomenon*, and *Dolichospermum* belong. Differentiation of heterocysts is a response to nutrient (nitrogen) stress and allows cyanobacteria to survive and exploit N-depleted Baltic Sea surface waters during the summer season. Further support of a connection is provided by data showing that several metacaspase genes present in *Anabaena* sp. PCC 7120 (Asplund-Samuelsson et al., 2012) are upregulated during heterocyst differentiation in response to nitrogen starvation (Flaherty et al., 2011; Mitschke et al., 2011). Also the expression of M32 is correlated to two heterocyst-related genes, albeit not to *hetR* (Table S4). Interestingly, heterocyst differentiation is a terminal differentiation process giving rise to non-dividing cells unable to propagate their DNA to the next generation (Maldener and Muro-Pastor, 2010), a process that in a sense mirrors that of PCD.

The transcriptome of *N. spumigena* CCY9414 exposed to high light and oxidative stress was recently analyzed (Kopf et al., 2015) and the upregulated genes indeed overlap to some extent with the M32 and M33 metacaspase gene expression clusters identified here (Table S5). Antisense RNA expression was also detected for all three metacaspase genes (Kopf et al., 2015; Figure 10), suggesting the presence of elaborate regulation circuitry. Transcription of M32 potentially starts from not less than four sites, if counting those inside the preceding gene (Kopf et al., 2015; Figure 10), emphasizing the importance of M32 expression and its classification as a house-keeping gene. Finally, in support of a role metacaspases and the development of multicellularity (cell differentiation, stresses) is the lack of metacaspases in unicellular cyanobacteria (Chroococcales), the numerically most common cyanobacteria in the Baltic Sea (Larsson et al., 2014).

### *Nodularia spumigena* metacaspase genes underline a putative connection to Sulfur metabolism

A novel connection between metacaspases and sulfur metabolism was suggested from metagenomic data (Figure S5) as well as the genomic context in *N. spumigena* (Figure 10). Explicitly, the M32-associated genes NSP_23620 and NSP_23600 are involved in sulfate assimilation (Prioretti et al., 2014) and methionine recycling (Parveen and Cornell, 2011), respectively. Two M33-coupled genes (DsbA-like thioredoxin and sulfite oxidase) are involved in formation of disulfide bonds (Shouldice et al., 2011) and their protection against sulfite induced damage (Kappler, 2011). Furthermore, the dehydrogenase and hydrolase associated with M31 are both involved in formaldehyde detoxification via a reaction depending on sulfur containing glutathione intermediates (van Straaten et al., 2009; Chen et al., 2012). Finally, as MsrB catalyzes the reduction of oxidized methionine residues (Weissbach et al., 2002), its association with M31 implies that this metacaspase may have a role related to protection against oxidative stress.

The FGE-sulfatase domain, capable of activating sulfatase enzymes (Appel and Bertozzi, 2015), is present in cyanobacterial metacaspases (Table 1), but not in *N. spumigena*, and widespread in bacterial metacaspases overall (Asplund-Samuelsson et al., 2012). Furthermore, the metagenomic contigs highlight a connection between metacaspases and sulfur in Bacteroidetes (Figure S5). Finally, many metacaspase genes identified here potentially belong to bacterial sulfur metabolism specialists such as *Desulfotalea, Desulfitobacterium, Desulfovibrio, Desulfococcus, Desulfobacula*, and *Desulfatibacillum* (Tables S1, S2). This further widens the connection of sulfur and metacaspases beyond *N. spumigena* and Cyanobacteria.

Activation of caspase-like proteases (i.e., detected by aspartate-specific proteolytic cleavage) in response to oxidative stress has been documented in laboratory experiments involving cultures or naturally harvested populations of the bloom-forming cyanobacterium *Trichodesmium erythraeum* (Berman-Frank et al., 2004) and natural populations (Ross et al., 2006) or cultures (Ding et al., 2012) of the bloom-forming cyanobacterium *Microcystis aeruginosa*. While sulfur compounds are implicated in both vulnerability to and protection against oxidative stress, not least via glutathione pathways (Battin and Brumaghim, 2009; Fahey, 2013), connections between metacaspases, sulfur metabolism, and oxidative stress now need to be experimentally verified.

### Metacaspases, programmed cell death, and cyanobacterial bloom dynamics in the Baltic Sea

In contrast to *Trichodesmium*, where metacaspase expression level is low prior to the onset of collapse of a mimicked bloom (Bar-Zeev et al., 2013), the metacaspase gene expression by Baltic Sea filamentous Cyanobacteria is not limited to a specific stage of the bloom. Rather, the genes are expressed throughout the season, which may be due to the potential role of metacaspases in house-keeping functions, to a continuous heterocyst differentiation as populations develop, or to PCD-related functions in a subset of the population at any given time. Indeed, the transient *Nodularia* population peak (25 July–15 August; Figure S2) did not correlate with any conspicuous metacaspase expression, as observed in laboratory cultures of *Trichodesmium*. Nevertheless, the expression of metacaspases M31 and M32 was correlated slightly to the relative population size (Figure 8B), which indicates a more subtle bloom-dependency.

Finally, yet another aspect related to cyanobacterial bloom collapse is the concurrent release of the toxin microcystin by the colony-forming *Microcystis aeruginosa* (Ross et al., 2006). Some Baltic Sea bloom-formers are notorious toxin producers. For instance, *N. spumigena* give rise to the hepatotoxic and carcinogenic compound nodularin, structurally similar to microcystin (Rinehart et al., 1988; Stolte et al., 2002). In this context it is interesting to note that the *N. spumigena* metacaspases M31 and M33 belong to gene expression clusters that include several nodularin toxin synthesis genes. This finding supports the existence of a connection between toxin biosynthesis, metacaspase expression and PCD. To what extent such a relationship exists may now be explored.

## Conclusions and future perspectives

Using “omics” approaches, we here provide evidence for a restricted distribution of metacaspases within the diverse microbial community of the Baltic Sea, but also support for metacaspases having important roles in multiple actors in the ecosystem. Metacaspases appear of particular importance to bacteria with larger cell size/genomes and elaborate life styles, including the heterocystous cyanobacterial radiation, which clearly dominates the expression of metacaspase genes. The data also support a functional diversification of metacaspases in these organisms, with potential roles related to nutrient stress, cell differentiation, and sulfur metabolism in connection to oxidative stress. The genetic background and the possible biochemical actions of the various types of metacaspases found here may now serve as platforms from which to experimentally define the physiological roles and explore the ecological impacts of metacaspases in nature.

## Author contributions

JA, JS, CD, KI, BB, and ME designed the experiments. JA, NC, and KI performed the sampling. JA, JS, CD, AA, JM, and NC carried out the analysis. JA, JS, CD, BB, KI, and ME interpreted the results. JA, JS, CD, BB, and ME drafted the manuscript. JA, JS, CD, AA, JM, NC, BB, KI, and ME revised the manuscript.

### Conflict of interest statement

The authors declare that the research was conducted in the absence of any commercial or financial relationships that could be construed as a potential conflict of interest.
