# Supplementary material for: Diversity and Expression of Bacterial Metacaspases in an Aquatic Ecosystem
Source: Front Microbiol. 2016 Jul 6;7:1043. doi: 10.3389/fmicb.2016.01043 (PMC4933709; doi:10.3389/fmicb.2016.01043)
Supplement: Supplementary file 9 [file Presentation1.PDF]

Supplementary Material

Diversity and expression of bacterial metacaspases in an aquatic ecosystem

Johannes Asplund-Samuelsson*, John Sundh, Chris L. Dupont, Andrew E. Allen, John P. McCrow, Narin Celepli, Birgitta Bergman, Karolina Ininbergs, Martin Ekman

* **Correspondence:** Johannes Asplund-Samuelsson: johannes.aspsam@gmail.com

1 Supplementary Data and Tables

The following supplementary data and tables are available separately:

Data_Sheet_S1_Metacaspase.refpkg.zip
Data_Sheet_S2_RecA.refpkg.zip
Data_Sheet_S3_Sequences_FASTA.zip

Table_S1_Sequences.xls
Table_S2_Transect_metacaspase_genera.xls
Table_S3_Statistical_tests.xls
Table_S4_Nodularia_hclust.xls
Table_S5_Nodularia_oxidative_stress.xls

2 Supplementary Figures

Supplementary Figures S1 through S7 are available on the following pages.

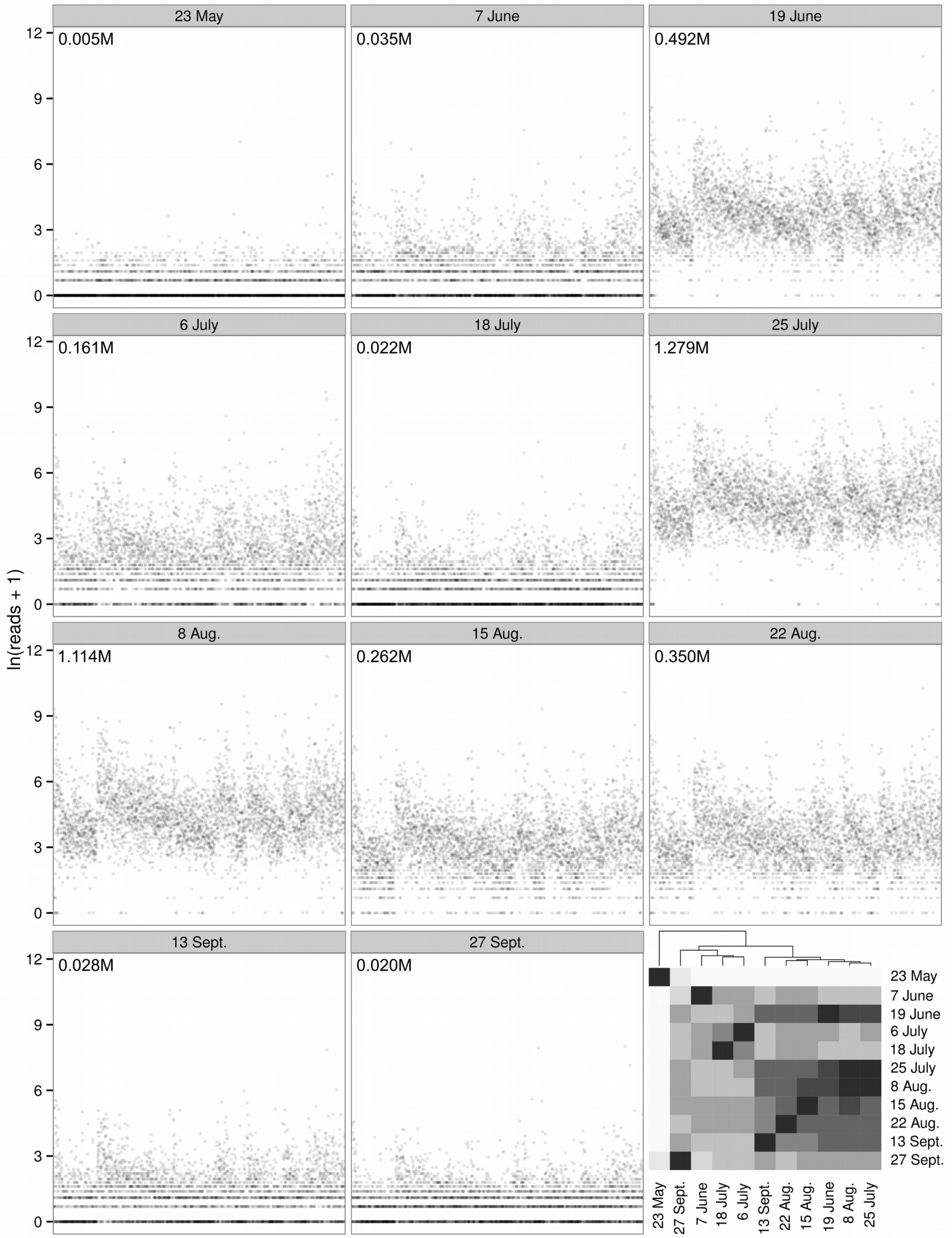

23 May
0.005M
7 June
0.035M
19 June
0.492M
6 July
0.161M
18 July
0.022M
25 July
1.279M
8 Aug.
1.114M
15 Aug.
0.262M
22 Aug.
0.350M
13 Sept.
0.028M
27 Sept.
0.020M
ln(reads + 1)
0
3
6
9
12
23 May
7 June
19 June
6 July
18 July
25 July
8 Aug.
15 Aug.
22 Aug.
13 Sept.
27 Sept.
23 May
27 Sept.
7 June
18 July
6 July
13 Sept.
22 Aug.
15 Aug.
19 June
8 Aug.
25 July

Figure S1. Illustration of the abundance and sequencing depth for *Nodularia spumigena* mRNA in the eleven metatranscriptomic samples from Askö in 2012 (Phase III). The 3 939 transcript open reading frames are arbitrarily distributed along the x-axis and the ln-transformed count numbers after adding one to all counts is displayed on the y-axis. Zero on the y-axis thus corresponds to zero counts. The total mapped read count in millions is shown in the upper left corner of each sample panel. The heatmap in the bottom right shows the pairwise Pearson correlation coefficient between samples. Columns are ordered according to the hierarchical clustering illustrated by the dendrogram. Rows are ordered by time, with the earliest sample of the season on the top. The minimum between-sample Pearson correlation coefficient is 0.25 and the maximum is 0.92. The darkest color corresponds to a coefficent of 1, which is obtained when correlating a sample to itself.

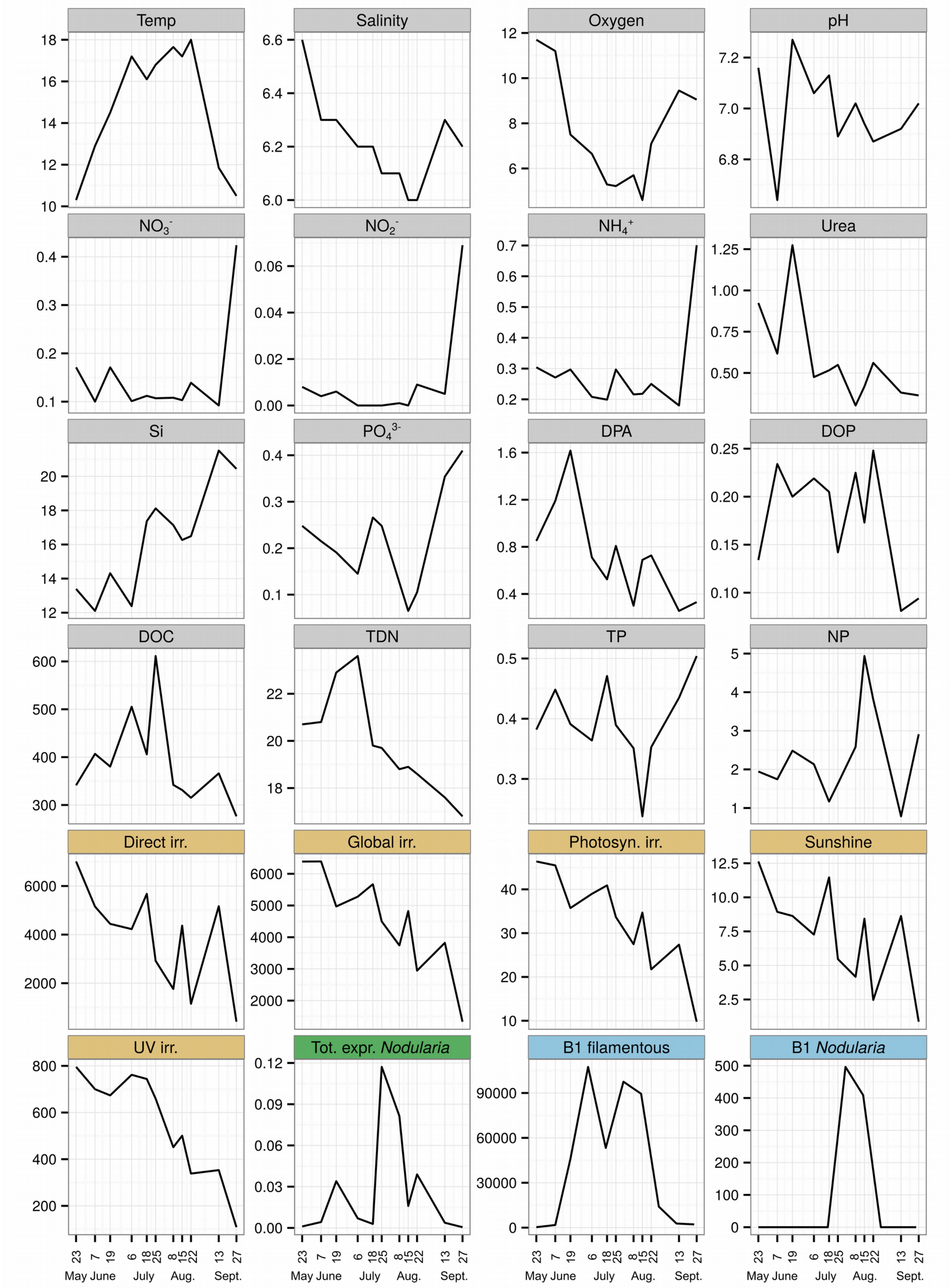

Temp
Salinity
Oxygen
pH
NO_3^-
NO_2^-
NH_4^+
Urea
Si
PO_4^{3-}
DPA
DOP
DOC
TDN
TP
NP
Direct irr.
Global irr.
Photosyn. irr.
Sunshine
UV irr.
Tot. expr. *Nodularia*
B1 filamentous
B1 *Nodularia*
23 7 19 6 18 25 8 15 22 13 27
May June July Aug. Sept.

Figure S2. Data describing the environment throughout the 2012 time series at the Askö sampling site (Phase III). Environmental variables measured on site (gray title background) include water temperature (°C), salinity (PSU), oxygen concentration (mg/L) and pH. The variables NO_3^-, NO_2^-, NH_4^+, urea, Si, PO_4^{3-}, dissolved primary amines (DPA), dissolved organic phosphate (DOP), dissolved organic carbon (DOC), total dissolved nitrogen (TDN) and total phosphate (TP), all given in µmol/L, were determined at Virginia Institute of Marine Science (http://web.vims.edu/admin/asc/). The calculated N/P ratio is also shown. Irraditation data (yellow) was acquired from the SMHI database STRÅNG (http://strang.smhi.se/) and is presented as rolling means of the three days leading up to and including the sampling day. Direct (Wh/m^2), global (Wh/m^2), photosynthetic (mol/m^2) and UV (mWh/m^2) irradiation as well as the number of sunshine hours is shown. Total expression of *Nodularia spumigena* relative to all mRNA (green) and monitoring data (blue) displaying the abundance (individuals per liter) of filamentous Cyanobacteria (orders Nostocales and Oscillatoriales) and *Nodularia* is shown in the bottom right corner. The monitoring data was downloaded from the SMHI database SHARK (Svenskt HavsARKiv). The data has been collected as a part of Swedish coordinated environmental monitoring by the Swedish Environmental Protection Agency (Naturvårdsverket).

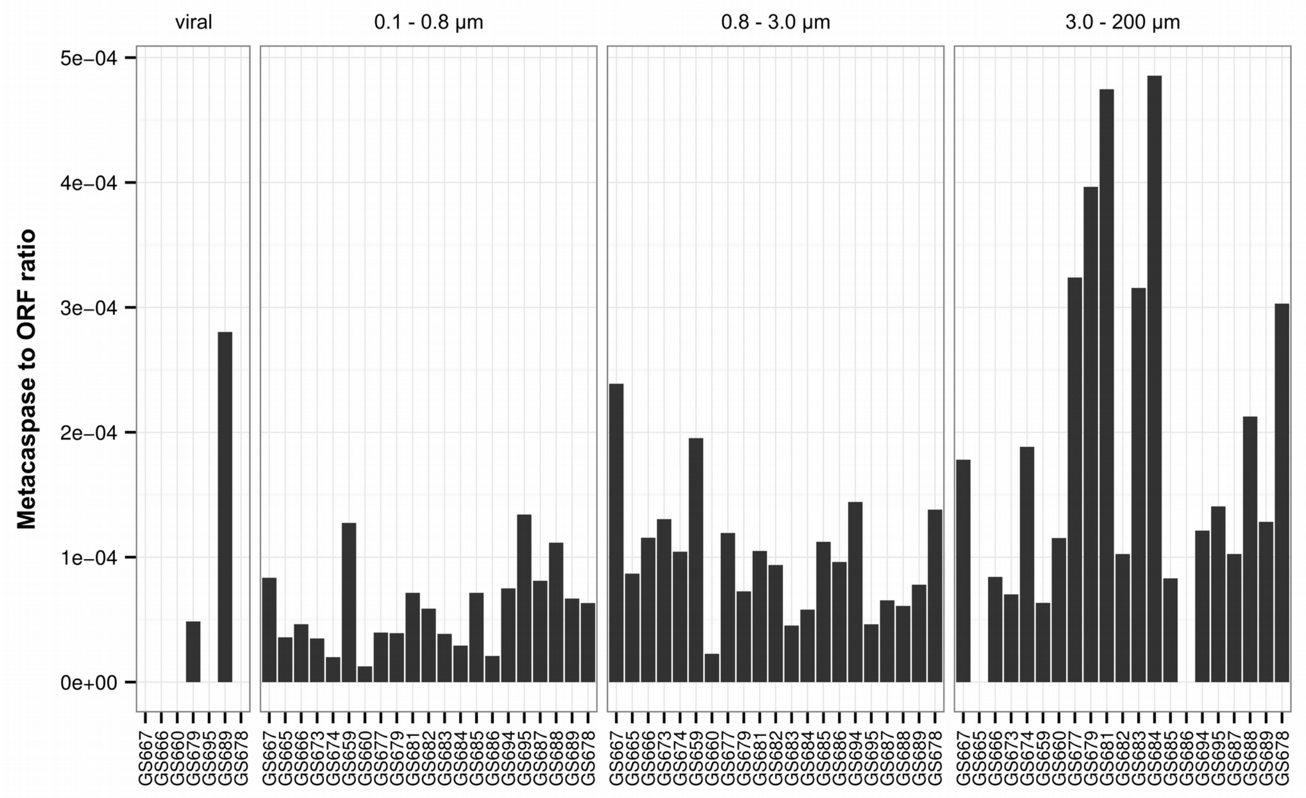

Figure S3. Bacterial metacaspase gene abundance in the 2009 transect (Phase I) metagenomes, relative to the total number of bacterial ORFs in all samples, from northern Sweden on the left (GS667; Lake Torne Träsk) to the Swedish west coast (GS689) on the right. The Landsort Deep samples are displayed on the far right (GS678). Values are shown in individual panels for each filter fraction.

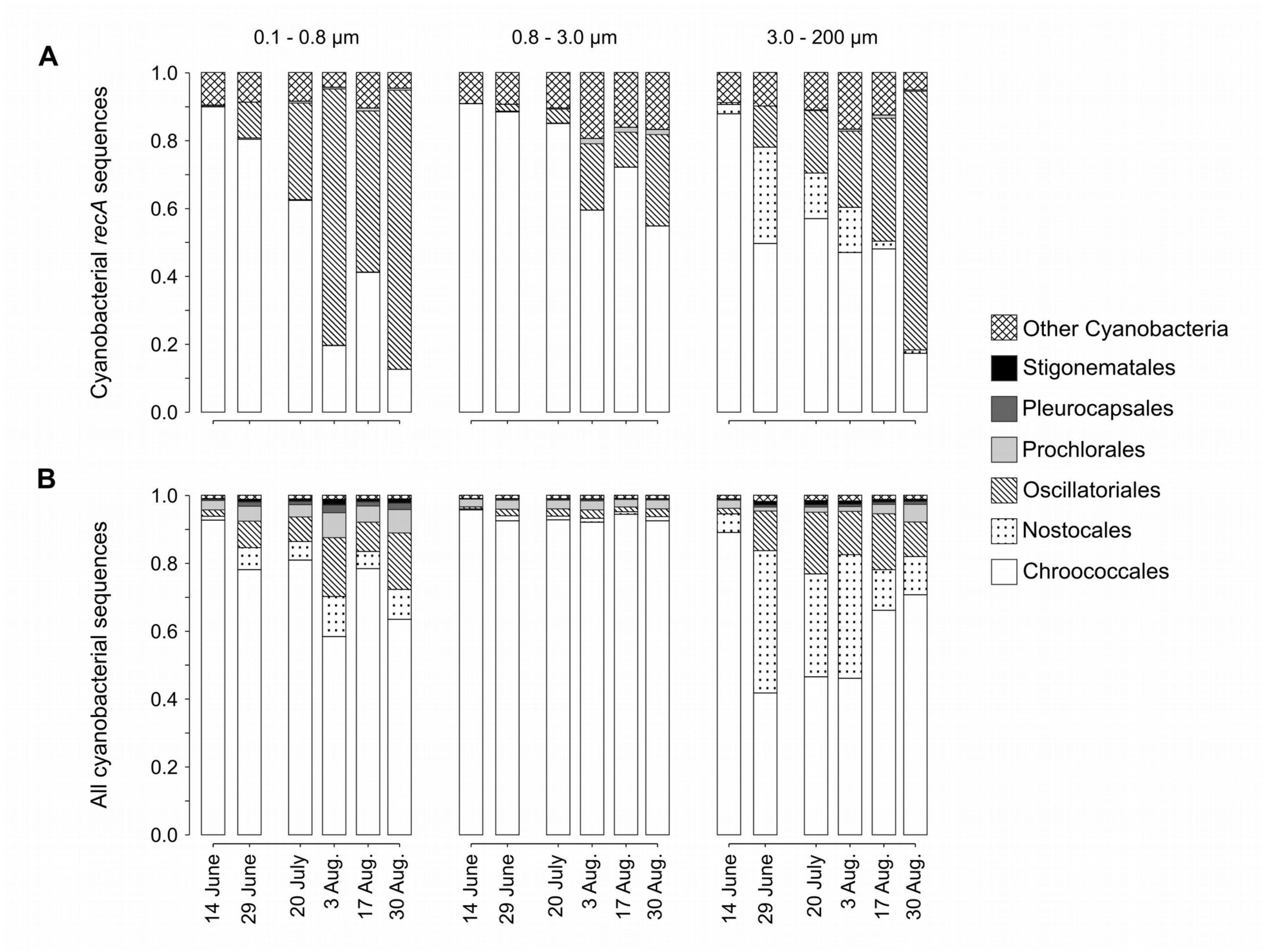

Figure S4. Relative abundances of cyanobacterial orders in the size fractions of the 2011 Askö time series metagenomes (Phase II), as identified by *recA* **(A)** and open reading frame **(B)** sequences. Values are summed average nucleotide coverages for the assembled contigs to which the identified genes belong. The *recA* sequences **(A)** and open reading frames **(B)** are taxonomically classified with pplacer (see Methods) and APIS, respectively. "Other Cyanobacteria" includes Gloeobacterales, unclassified Cyanobacteria, environmental sample Cyanobacteria and sequences that were not classified at order level or better.

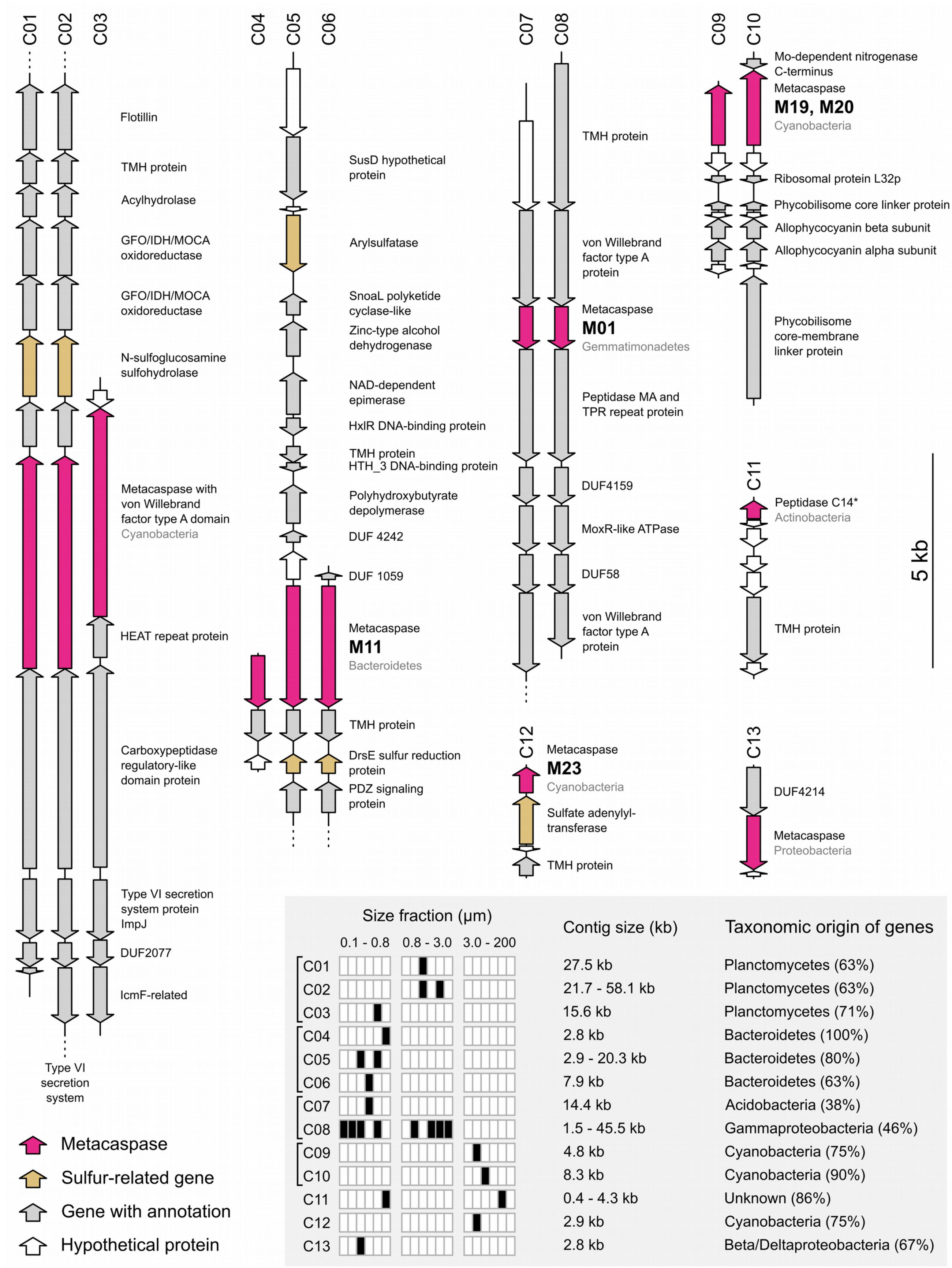

C01
C02
C03
C04
C05
C06
C07
C08
C09
C10
Flotillin
TMH protein
Acylhydrolase
GFO/IDH/MOCA oxidoreductase
GFO/IDH/MOCA oxidoreductase
N-sulfoglucosamine sulfohydrolase
Metacaspase with von Willebrand factor type A domain
Cyanobacteria
HEAT repeat protein
Carboxypeptidase regulatory-like domain protein
Type VI secretion system protein ImpJ
DUF2077
IcmF-related
Type VI secretion system
SusD hypothetical protein
Arylsulfatase
SnoaL polyketide cyclase-like
Zinc-type alcohol dehydrogenase
NAD-dependent epimerase
HxlR DNA-binding protein
TMH protein
HTH_3 DNA-binding protein
Polyhydroxybutyrate depolymerase
DUF 4242
DUF 1059
Metacaspase
M11
Bacteroidetes
TMH protein
DrsE sulfur reduction protein
PDZ signaling protein
TMH protein
von Willebrand factor type A protein
Metacaspase
M01
Gemmatimonadetes
Peptidase MA and TPR repeat protein
DUF4159
MoxR-like ATPase
DUF58
von Willebrand factor type A protein
Mo-dependent nitrogenase C-terminus
Metacaspase
M19, M20
Cyanobacteria
Ribosomal protein L32p
Phycobilisome core linker protein
Allophycocyanin beta subunit
Allophycocyanin alpha subunit
Phycobilisome core-membrane linker protein
C11
Peptidase C14*
Actinobacteria
TMH protein
5 kb
C12
Metacaspase
M23
Cyanobacteria
Sulfate adenylyl-transferase
TMH protein
C13
DUF4214
Metacaspase
Proteobacteria
Metacaspase
Sulfur-related gene
Gene with annotation
Hypothetical protein
Size fraction (µm)
0.1 - 0.8
0.8 - 3.0
3.0 - 200
Contig size (kb)
Taxonomic origin of genes
C01 27.5 kb Planctomycetes (63%)
C02 21.7 - 58.1 kb Planctomycetes (63%)
C03 15.6 kb Planctomycetes (71%)
C04 2.8 kb Bacteroidetes (100%)
C05 2.9 - 20.3 kb Bacteroidetes (80%)
C06 7.9 kb Bacteroidetes (63%)
C07 14.4 kb Acidobacteria (38%)
C08 1.5 - 45.5 kb Gammaproteobacteria (46%)
C09 4.8 kb Cyanobacteria (75%)
C10 8.3 kb Cyanobacteria (90%)
C11 0.4 - 4.3 kb Unknown (86%)
C12 2.9 kb Cyanobacteria (75%)
C13 2.8 kb Beta/Deltaproteobacteria (67%)

Figure S5. Illustration of 2011 time series (Phase II) metagenomic contigs that contain open reading frames (ORFs) coding for metacaspases. Gene names and putative functions are based on KEGG, Pfam and TMHMM annotations. A protein product with only transmembrane helices, identified by TMHMM, is indicated by the tag "TMH protein". Metacaspases from the metatranscriptome that align to the metagenomic ORFs are indicated in bold (M01, >99% identity; M11, 100%; M19, 100%; M20, 87.5%; M23, 67.5%). The light gray text gives the phylum for the metacaspase, as classified by pplacer. The displayed contigs C01-C13 have at least three ORFs, carry a bacterial metacaspase and are the largest representatives of 99% identity clusters of one or more contigs. The sample of origin for the contigs in these clusters are shown in the table in the bottom right corner. There, the rectangles represent the different size fractions and the six smaller boxes within represent each of the sampling dates, *i.e.* 14 June, 29 June, 20 July, 3 August, 17 August and 30 August. Additionally, the table lists sizes of the contigs and the APIS organism group of origin for the majority of the ORFs on the representative contig, *i.e.* C01-C13, as a percentage of all ORFs on the contig.

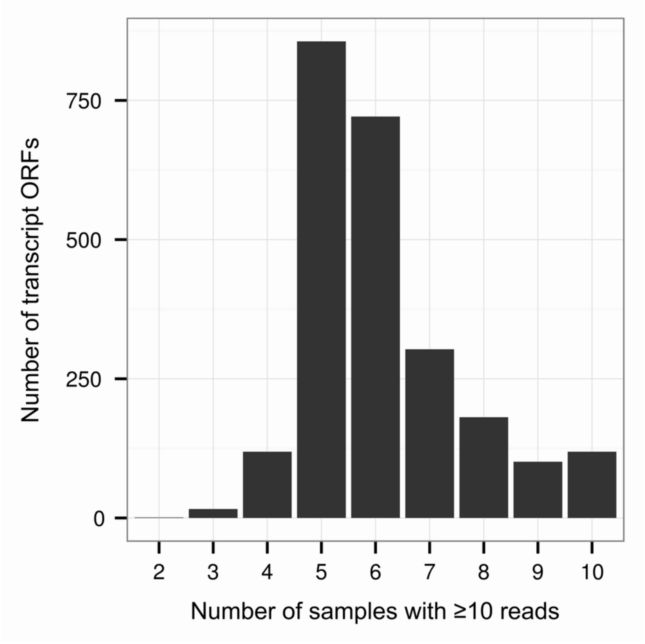

Figure S6. Coverage of metatranscriptome transcripts (open reading frames; ORFs) identified as *Nodularia spumigena* CCY9414, in the Phase III (2012) sampling season. The coverage is described as the number of samples in which each transcript ORF has 10 or more mapped reads (x-axis). The number of transcript ORFs in each category is shown on the y-axis. The data is based on the 2 417 ORFs remaining after filtration, *i.e.* requiring a total of ≥200 reads in the 10 samples under consideration, and no more than three zero counts among these samples.

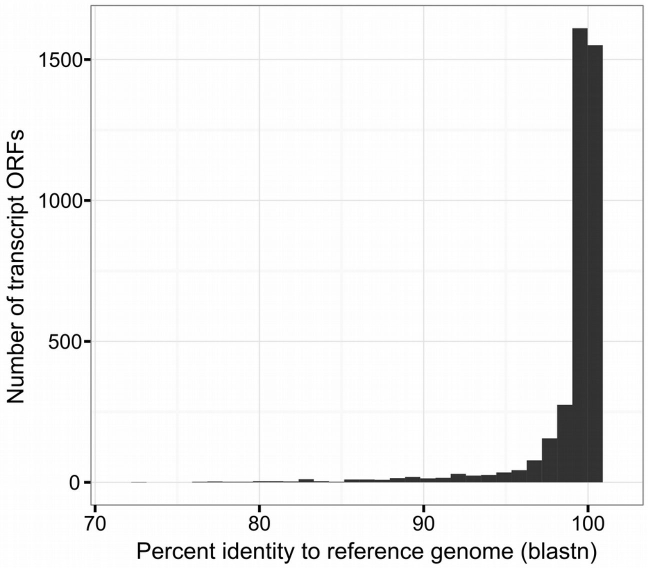

Figure S7. Percent identity of blastn hits using metatranscriptome transcripts (open reading frames; ORFs) identified as *Nodularia spumigena* CCY9414 as queries and the corresponding sequenced genome as the target (GenBank accession CP007203.2). Only the highest scoring blastn hit was accepted. The first hit in the list was chosen in the 26 cases of multiple hits at the same score. 156 (3.8%) transcripts did not yield a blastn hit versus the sequenced genome.
